# Genetic parameters of drinking and feeding traits of wean-to-finish pigs under a polymicrobial natural disease challenge

**DOI:** 10.1186/s40104-021-00622-x

**Published:** 2021-09-08

**Authors:** Jian Cheng, Austin M. Putz, John C. S. Harding, Michael K. Dyck, Frederic Fortin, Graham S. Plastow, Pig Gen Canada, Jack C. M. Dekkers

**Affiliations:** 1 Department of Animal Science, Iowa State University, Ames, IA 50011 USA; 2 Hendrix Genetics, Swine Business Unit, Boxmeer, The Netherlands 5831 CK; 3 Department of Large Animal Clinical Science, University of Saskatchewan, Saskatoon, SK S7N 5A2 Canada; 4 Department of Agriculture, Food and Nutritional Science, University of Alberta, Edmonton, AB T6G 2R3 Canada; 5 Centre de Développement du Porc du Québec Inc., Québec City, G1V 4M6 Canada; 6 PigGen Canada Research Consortium, Guelph, Ontario N1H4G8 Canada

**Keywords:** Disease resilience, Feeding and drinking behavior, Genetic parameters, Pigs

## Abstract

**Background:**

The pork industry faces unprecedented challenges from disease, which increases cost of production and use of antibiotics, and reduces production efficiency, carcass quality, and animal wellbeing. One solution is to improve the overall resilience of pigs to a broad array of common diseases through genetic selection. Behavioral changes in feeding and drinking are usually the very first clinical signs when animals are exposed to stressors such as disease. Changes in feeding and drinking behaviors in diseased pigs may reflect the way they cope with the challenge and, thus, could be used as indicator traits to select for disease resilience. The objectives of this study were to estimate genetic parameters of feeding and drinking traits for wean-to-finish pigs in a natural polymicrobial disease challenge model, to estimate genetic correlations of feeding and drinking traits with growth rate and clinical disease traits, and to develop indicator traits to select for disease resilience.

**Results:**

In general, drinking traits had moderate to high estimates of heritability, especially average daily water dispensed, duration, and number of visits (0.44 to 0.58). Similar estimates were observed for corresponding feeding traits (0.35 to 0.51). Most genetic correlation estimates among drinking traits were moderate to high (0.30 to 0.92) and higher than among feeding traits (0 to 0.11). Compared to other drinking traits, water intake duration and number of visits had relatively stronger negative genetic correlation estimates with treatment rate and mortality, especially across the challenge nursery and finisher (− 0.39 and − 0.45 for treatment rate; − 0.20 and − 0.19 for mortality).

**Conclusion:**

Most of the recorded drinking and feeding traits under a severe disease challenge had moderate to high estimates of heritability, especially for feed or water intake duration and number of visits. Phenotypic and genetic correlations among the recorded feeding traits under disease were generally low but drinking traits showed high correlations with each other. Water intake duration and number of visits are potential indicator traits to select for disease resilience because of their high heritability and had moderate genetic correlations with treatment and mortality rates under severe disease.

**Supplementary Information:**

The online version contains supplementary material available at 10.1186/s40104-021-00622-x.

## Introduction

Pork is the most consumed animal protein in the world and accounts for about 32% of total meat consumption [1]. The demand for animal protein is growing and is expected to increase by 73% by 2050 [2]. However, the pork industry faces unprecedented challenges from diseases such as porcine reproductive and respiratory syndrome, porcine epidemic diarrhea, African swine fever, swine influenza, and others. Exposure to disease reduces production efficiency and carcass quality, increasing cost of production and use of antibiotics, and reducing animal wellbeing. Holtkamp et al. [3] estimated the cost of porcine reproductive and respiratory syndrome in the U.S. at $664 million per year. Paarlberg [4] reported that in the U.S., the porcine epidemic diarrhea virus outbreak in 2013 cost between $900 million and $1.8 billion. Antibiotics, vaccines, and biosecurity or management procedures are the main measures used for disease control but they are not always effective and vaccines are disease specific. An alternative is to improve the overall resilience of pigs to a broad array of common diseases through genetic selection [5, 6]. Disease resilience is defined as the ability of an animal to maintain performance in the face of pathogen exposure [7]. Disease resilience is, however, difficult to incorporate in breeding programs because nucleus breeding stock must be raised in high-health conditions, preventing the collection of disease resilience data. To collect data on disease resilience and study the genetic basis of response of pigs to multiple diseases, a natural polymicrobial disease challenge model was established at a research station in Quebec, Canada [8]. Estimates of genetic parameters of production and clinical disease data from this model were reported by Putz et al. [8] and Cheng et al. [9].

Behavioral changes in feeding and drinking are one of the first observable clinical signs when animals are exposed to stress such as disease or extreme temperatures. Berghof et al. [7] explored opportunities to determine new resilience indicators based on longitudinal data. Putz et a1. [8] and Cheng et al. [9] showed that phenotypes derived from a pig’s feeding behavior under a disease challenge, including day-to-day variation and the proportion of off-feed days derived from the individual feed intake and duration data, are genetically correlated with disease resilience. A pig’s drinking behaviors may also change when affected by disease [10–12]. The amount of water each pig drinks and drinking behaviors such as the number of visits to the trough or other drinking systems, and duration of drinking on a daily basis or across the test period can vary significantly with disease and stress levels, as well as with temperature, humidity, and diet [13]. Specifically, Dybkjaer et al. [14] reported that diarrhea in young pigs could be detected about one day before clinical signs were apparent by monitoring water usage. Ahmed et al. [15] reported that Salmonella infection in pigs resulted in reduced feeding and drinking activity. Kruse et al. [11] used the wavelet transform to analyze water intake patterns to differentiate healthy and non-healthy sows. Changes in drinking behaviors in diseased pigs may reflect the way they cope with or are impacted by the pathogen, thereby indicating the health status, and may be partly influenced by the genetics of the pig [16].

The objectives of this study were to: 1) estimate genetic parameters for feeding and drinking traits for wean-to-finish pigs from a natural disease challenge model; 2) estimate the genetic relationship of feeding and drinking traits with growth rate and clinical disease traits; 3) evaluate the usefulness of day-to-day variation and proportion of off days derived from drinking traits as indicators of disease resilience; and 4) develop other drinking and feeding indicator traits to select for disease resilience. For this study, the growth, feeding, and clinical disease data analyzed by Cheng et al. [9] were used but the focus here was on drinking behavior traits that were not analyzed previously. Results for some feed intake and behavior traits from Cheng et al. [9] are reported here for comparison with results obtained here for water intake and behavior traits.

## Materials and methods

This study was carried out in accordance with the Canadian Council on Animal Care guidelines (CCAC; https:((www.ccac.ca(en(certification(about-certification). The protocol was approved by the Protection Committee of the Centre de Recherche en Sciences Animales de Deschambault (CRSAD) and the Animal Care and Use Committee at the University of Alberta (AUP00002227). The project was fully overseen by the Centre de développement du porc du Québec (CDPQ) in Québec, Canada, and its herd veterinarian together with project veterinarians.

### Data collection

All data and samples were collected by trained research staff from CDPQ using established natural challenge protocols, as described by Cheng et al. [9]. Data on 3,285 Large White by Landrace barrows from seven breeding companies were available. The natural challenge protocol consisted of three phases: (1) quarantine nursery (19 d on average, beginning at 3 weeks of age); (2) challenge nursey (27 d on average); and (3) finishing phase (100 d on average). The average group sizes in the three phases were 4.25, 7.16, and 10.72 pigs per pen, respectively. Pigs were re-grouped when moved to the challenge nursery and to the finisher.

Details of the most phenotypes that were recorded and analyzed in the challenge nursery and finisher were described in Cheng et al. [9] and included body weights, individual medical treatments, mortality, health scores, feed intake, and carcass traits. Health scores were assigned by trained personnel based on clinical signs on a 1 to 5 scale: 1 = severe clinical signs with wasting and 5 = in perfect health, as described by Cheng et al. [9]. Treatment rates of individual pigs were adjusted by multiplying the number of individual treatments a pig received in the corresponding phase by the ratio of the average length of that phase and the number of days the pig spent in that phase. Mortality was recorded as 0 = survived and 1 = died. For treatment rates and growth rate, data from pigs that died in the finisher were included in some analyses, with imputation and expansion of treatment and growth rates, as described in Cheng et al. [9].

Individual feed (dry pellets) intake data were recorded in the finishing barn using IVOG feeding stations (Insentec, Marknesse, Netherlands), with pig recognition using a radio frequency identification system. Daily feed intakes that were negative or greater than 4 kg were removed and, then, individual visit data were further edited using the methods of Casey et al. [17]. Individual drinking data were also recorded in the finishing barn using a single-space individual water intake recording system (Fig. S1) for each pen. Designed and developed by CDPQ staff, the system allowed the recording of water intake and associated data for each pig’s visit to the drinker, with identification of pigs using the radio frequency identification system. The water delivery system included a 3 L bowl that was closed on 3 sides to reduce water waste, a water nipple in the bowl that can be activated by the pigs, a water meter on the line that feeds the nipple, and a water level meter for the bowl. For each visit, the system recorded the time of day, the duration, and the amount of water dispensed from the nipple (dispensed) and removed from or added to the bowl. Water disappearance for each visit was calculated as water dispensed plus the change in water level in the bowl (level at the start of the visit - level at the end of the visit). For drinking and feeding traits, only data on pigs that survived to slaughter were included for analyses to avoid the potential impact of having intake data only over a shorter period. For feed intake, average daily feed intake, duration, and number of visits were analyzed. In total, feeding data for 2,337 pigs were included in analyses, while drinking data on 2,331 pigs were available before data cleaning.

All animals were genotyped with the 650 k Affymetrix Axiom Porcine Genotyping Array by Delta Genomics (Edmonton AB, Canada). The 435,172 SNPs that passed quality control, as described by Cheng et al. [9], were utilized for analysis.

### Drinking behavior data editing

Only drinking data collected between 70 and 150 days of age were used for each pig. In addition, to eliminate the potential impact of pen density on drinking traits, only data that were collected before the first pigs from a batch were sent to slaughter were used for analysis. Water disappearance data were summed across all visits on a day to compute the water disappearance and duration for each pig for each day. Water disappearance rate was also computed for each day and each pig as the ratio of water disappearance and intake duration. For number of visits, visits of the same pig that were separated by less than 10 s were combined into one visit and visits with zero water consumption were not counted as visits. Because water dispensed was more heritable than water disappearance (0.44 versus 0.34, see later), water dispensed and dispense rate were also computed for each day and each pig, i.e. ignoring changes in water level from the bowl.

Representative drinking and feeding data for a randomly selected pig are shown in Figs. S2-S5, illustrating the variability in daily water intake data, which was much larger than for feed intake data, in part because of the technical difficulty of measuring water intake. To reduce the effect of measurement errors, very strict editing protocols were implemented for the drinking data. Since reasons for data errors are expected to be technical in nature, we expect data errors to be independent of the health status of the pig, such that removing apparent error data is not expected to bias results but reduce noise in the data. And indeed, the editing steps increased estimates of heritability for all traits by 52% to 100%, suggesting that the edits removed data that contained a lot of noise.

**Fig. 1:**
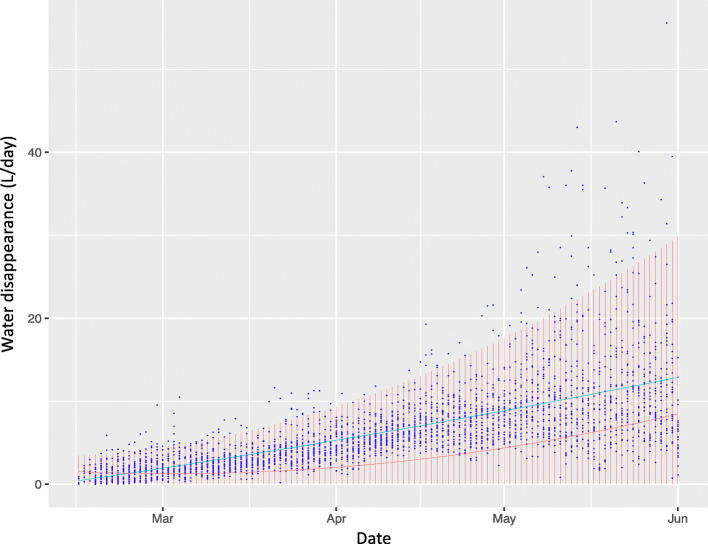
Observed water disappearance by day for a random batch of pigs (blue dots). The vertical red bars represent the predicted mean (red line) + 2 times the predicted interquartile range. The blue line is predicted interquartile range. Observations outside the red bars are considered outliers

To implement the editing steps, firstly, for each drinking trait defined above, outliers were identified on a daily basis within each batch if a pig’s data for that day was greater than the predicted mean + 2 times the predicted interquartile range (IQRP) for that day and batch. The predicted mean was based on linear-quadratic regression of the drinking trait data on date by batch, as shown in Fig. 1, while the IQRP was based on linear-quadratic regression on date of the IQR by day for that batch. A pig’s data for a given day were removed for all drinking traits if an outlier was detected for one or more drinking traits for that day. This resulted in removal of all drinking data for 9% of days across all pigs (different pigs can have a different number of days removed). Secondly, because of the importance of having data at the start and end of the test period, all data on pigs with more than 10 d with missing data at the start (around 70 to 90 days of age) or at the end (around 130 to 150 days of age) of the finisher period were removed by trait. This removed 32% to 41% of pigs, depending on the trait, and is another reason why only pigs that survived to slaughter were used in this study.

### Derivation of drinking phenotypes

After outlier removal, the following phenotypic random regression model was used to predict the phenotype for a given drinking trait for each pig for each day:

1 $${y}_{ijk}= Batc{h}_i+{b}_1\ast Ag{e}_{ijk}+{b}_2\ast Ag{e}_{ijk}^2+ Pe{n}_j+{\sum}_{l=0}^2{a}_{ijk l}\ast Ag{e}_{ijk}^l+{e}_{ijk}$$

where *y*_*ijk*_ is the drinking trait phenotype; *Batch*_*i*_ is a fixed batch effect (*i* = 1, …, 50), which also accounted for the effect of company because company was confounded with batch; *Age*_*ijk*_ is the entry age; *b*_*1*_ and *b*_*2*_ are fixed regression coefficients; *Pen*_*j*_ is the random effect of finisher pen within batch, with vector $\boldsymbol{Pen}\sim N\left(\mathbf{0},{\boldsymbol{I}\sigma}_P^2\right)$, where ${\sigma}_P^2$ is the pen variance and ***I*** is the identity matrix; ${\sum}_{l=0}^2{a}_{ijk l}\ast Ag{e}_{ijk}^l$ is the random regression on age for pig *k*, where *a*_*ijkl*_ denotes the random regression coefficients for the *k*^th^ animal and *l* is the order of the polynomial (*l* = 0,1, and 2), with variance-covariance structure for the random regression coefficients for an individual equal to $$Var\left[\begin{array}{c}{a}_{ijk0}\\ {}{a}_{ijk1}\\ {}{a}_{ijk2}\end{array}\right]=\left[\begin{array}{ccc}{\sigma}_0^2& {\sigma}_{0,1}& {\sigma}_{0,2}\\ {}{\sigma}_{0,1}& {\sigma}_1^2& {\sigma}_{1,2}\\ {}{\sigma}_{0,2}& {\sigma}_{1,2}& {\sigma}_2^2\end{array}\ \right]$$, where ${\sigma}_0^2,{\sigma}_1^2, and\ {\sigma}_2^2$ are the variances for the intercept, linear, and quadratic regression coefficient, respectively, and *σ*_0, 1_, *σ*_0, 2_, and *σ*_1, 2_ are the corresponding covariances; *e*_*ijk*_ is the residual effect, with vector $\boldsymbol{e}\sim N\Big(\mathbf{0},{\boldsymbol{I}\sigma}_e^2$), where ${\sigma}_e^2$ is the residual variance, allowing for heterogeneous residual variances by age class: 53 to 82, 83 to 112, 113 to 142, 143 to 172, and greater than 172 days of age. Investigation of the optimal division in age classes was beyond the scope of this study.

**Fig. 2:**
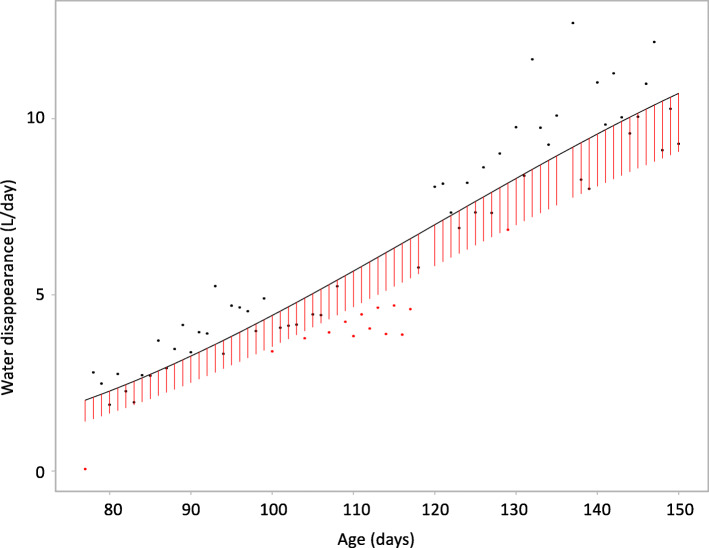
Derivation of day-to-day variation and the proportion of off-water days for the water disappearance data of an randomly selected pig. Dots are the observed water disappearance by day. Solid black line represents the predicted water disappearance for this pig based on a quadratic random regression model; day-to-day variation in water intake was computed as the square root of the sum of squared residuals. Red vertical lines represent the predicted interquartile range of random regression residuals within batch based on a quadratic regression model; observations that had residuals less than the predicted interquartile range were considered off-water days (red dots)

Average daily drinking phenotypes were computed based on the predicted values from the random regression model and included average daily water dispensed (ADWD), average daily water disappearance (ADWI), average daily water intake duration (WIDUR), average daily number of water intake visits (WInVisits), average daily water dispensed rate (WDRT), and average daily water disappearance rate (WIRT). Day-to-day variation in drinking phenotypes was computed for each pig as the mean square root of deviations of observed from predicted values from the random regression model (Fig. 2) and included day-to-day variation in water dispensed (VAR_WD_), water disappearance (VAR_WI_), water intake duration (VAR_WIDUR_), number of water intake visits (VAR_WInVisits_)_,_ water dispense rate (VAR_WDRT_), and water disappearance rate (VAR_WIRT_)_._ The coefficient of variation for day-to-day variation phenotypes were calculated as the square root of the mean of the day-to-day variation of the drinking trait divided by the mean of corresponding average daily drinking trait across pigs, e.g. $\sqrt{{\mathrm{VAR}}_{WI}}/\mathrm{ADWI}$. A day for a pig was considered an off-water day for a given drinking trait if the residual for that day from the random regression model was less than the IQRP of residuals (Fig. 2), where the IQRP was obtained for each day and each batch using linear quadratic regression of the observed IQR of residuals on date. The proportion of off-water days for each drinking trait was computed as the number of off-water days divided by total number of days with data and included the proportion of off-water days based on water dispensed (OFF_WD_), water disappearance (OFF_WI_), water intake duration (OFF_WIDUR_), number of water intake visits (OFF_WInVisits_)_,_ water dispense rate (OFF_WDRT_), and water disappearance rate (OFF_WIRT_). For pigs with more than 30% off-water days for a given drinking trait all data for that trait were removed because their data likely reflects a malfunction of the water drinking or identification system rather than poor resilience. This resulted in removal of 0.2%, 5%, 18%, 18%, 5%, and 7% of pigs for OFF_WInVisits_, OFF_WIDUR_, OFF_WI_, OFF_WD_, OFF_WIRT_, and OFF_WDRT_, respectively.

To determine whether off-water days coincided with off-feed days, a pig’s off-water days were compared with that pig’s off-feed days throughout the finishing period for days for which a pig had both feeding and drinking data. In contrast to off-water intake days, off-feed intake days were identified based on quantile regression, as described by Putz et al. [8]. To explore the relationship between off days and health treatments, overlaps of off-water or off-feed days with days in which the pig received treatment were also investigated. To allow for some difference in the timing of off-water, off-feed, and treatment days, a 7-day rolling window was also used to identify the coincident off-feed, off-water, and treatment days. For example, off-water and off-feed events were considered to overlap for a 7-day window if that window included at least one off-water and one off-feed day. A similar strategy was used for overlaps with treatments. Chi-square tests were used to determine whether off-water, off-feed, and treatment events coincided significantly more than expected. The expected number of overlapping off-feed and off-water days was computed as the products of the proportion of off-feed days, the proportion of off-water days, and the total number of days with feeding or drinking data (92,682). The expected numbers of coincident off-feed or off-water days with treatment were computed as the products of the proportion of off-feed or off-water days, the proportion of treatment days, and the total number days with feeding or drinking data. When using 7-day windows, proportions and numbers of days were replaced by proportions and numbers of windows with at least one off-water, off-feed, or treatment day.

### Variance component estimation

Variance components were estimated by genomic best linear unbiased prediction (GBLUP) using ASReml 4.0 [18]. The following general model was used in single-trait and bivariate analyses to estimate variance components and genetic correlations:

2 $${y}_{ijk l}= Batc{h}_i+ Ag{e}_{ijk l}+ Pe{n}_j+{\mathrm{u}}_{ijk l}+ litte{r}_{ijk}+{e}_{ijk l}$$

where *y*_*ijkl*_ is the trait; *Batch*_*i*_ is a fixed batch effect (i = 1, …, 50); *Age*_*ijkl*_ is the covariate of age when the pig entered the quarantine nursery; *Pen*_*j*_ is the random effect of pen by batch corresponding the different phases, with vector $\boldsymbol{Pen}\sim \mathrm{N}\left(\mathbf{0},{\boldsymbol{I}\sigma}_P^2\right)$, where ${\sigma}_P^2$ is the pen variance; *u*_*ijkl*_ is the random additive genetic effect, with the vector $\boldsymbol{u}\sim \mathrm{N}\Big(\mathbf{0},\boldsymbol{G}{\sigma}_A^2$), where ***G*** is the genomic relationship matrix and ${\sigma}_A^2$ is the additive genetic variance; *litter*_*ijk*_ is the litter environmental effect, with vector $\boldsymbol{litter}\sim \mathrm{N}\Big(\mathbf{0},{\boldsymbol{I}\sigma}_l^2$), where ${\sigma}_l^2$ is the litter environmental variance; *e*_*ijkl*_ is the residual effect, with vector $\boldsymbol{e}\sim \mathrm{N}\Big(\mathbf{0},\boldsymbol{I}{\sigma}_e^2$), where ${\sigma}_e^2$ is the residual variance. The genomic relationship matrix, ***G***, was created separately for each company using the software preGSf90 [19] based on method one of VanRaden [20]. Then, the seven ***G*** matrices were combined into one ***G*** matrix, with genetic relationships between companies set to zero, such that the analyses focused on pooled within-company variances. A discussion of the use of the within-company versus across company relationship matrix is in Cheng et al. [9]. Water-to-feed and feed-to-water ratio traits were also analyzed as conditional traits by fitting the corresponding feeding or drinking phenotype of that animal as a covariate. For example, for analysis of ADFI conditional on ADWI, ADWI was fitted as a covariate in the model for analysis of ADFI to remove the effect of ADWI on ADFI.

## Results

### Phenotypic data

Table 1 shows phenotypic means (SD) for all drinking and feeding traits, and the numbers of records retained for analysis by trait. Comparing means for feeding and drinking phenotypes, pigs spent more than four times as much time eating than drinking on a daily basis but made nearly twice as many visits to the drinker than to the feeder per day. As a result, pigs spent seven times more time eating than drinking per visit. Day-to-day variation in feed intake duration was nearly three times greater than day-to-day variation in water intake duration, while day-to-day variation for the number of feed intake visits was less than for the number of water intake visits. Day-to-day variation in daily feed intake and in feed intake rate was lower than for water disappearance and water disappearance rate, respectively. However, the coefficients of day-to-day variation were greater for feed intake, number of visits, and feed intake rate than for the corresponding drinking traits. The proportions of off-water or off-feed intake days were small but twice as large for drinking than for feeding traits, noting that different methods were used to identify off-water versus off-feed intake days.

**Table 1:** Summary statistics and estimates (SE in parentheses) of heritability (h^2^) and litter effects (c^2^) for drinking and feeding traits in the finisher

Trait	No. records	Mean	SD	CV	h^2^ (SE)	c^2^ (SE)
***Drinking***						
ADWI, L/d	1,379	5.06	2.42	0.48	0.34 (0.08)	0.05 (0.04)
ADWD, L/d	1,426	4.84	3.30	0.68	0.44 (0.07)	0.04 (0.04)
WIDUR, min/d	1,525	14.73	4.37	0.30	0.54 (0.07)	0.06 (0.03)
WInVisits	1,598	31.32	7.49	0.24	0.58 (0.06)	0.01 (0.03)
WIRT, L/min	1,529	0.36	0.13	0.36	0.25 (0.06)	0.00 (0.00)
WDRT, L/min	1,533	0.32	0.18	0.56	0.30 (0.07)	0.02 (0.03)
ADWI|ADFI	1,379	–	–	–	0.42 (0.08)	0.02 (0.04)
ADWD|ADFI	1,426	–	–	–	0.50 (0.07)	0.02 (0.03)
WIDUR|FIDUR	1,525	–	–	–	0.56 (0.07)	0.05 (0.03)
WInVisits|FInVisits	1,598	–	–	–	0.60 (0.06)	0.02 (0.03)
***Feeding***						
ADFI, kg/d	2,337	2.20	0.32	0.15	0.35 (0.05)	0.03 (0.03)
FIDUR, min/d	2,337	59.51	11.49	0.19	0.49 (0.06)	0.04 (0.03)
FInVisits	2,337	17.71	6.79	0.38	0.51 (0.05)	0.08 (0.03)
FIRT, kg/min	2,337	0.038	0.009	0.25	0.45 (0.05)	0.03 (0.03)
ADFI|ADWI	2,337	–	–	–	0.42 (0.06)	0.00 (0.00)
ADFI|ADWD	2,337	–	–	–	0.42 (0.06)	0.00 (0.00)
FIDUR|WIDUR	2,337	–	–	–	0.55 (0.07)	0.02 (0.03)
FInVisits|WInVisits	2,337	–	–	–	0.46 (0.07)	0.12 (0.04)
***Drinking***						
VAR_WI_	1,379	2.08	1.41	0.29^1^	0.35 (0.07)	0.00 (0.00)
VAR_WD_	1,426	2.51	1.66	0.33^1^	0.38 (0.08)	0.01 (0.04)
VAR_WIDUR_	1,525	4.84	2.06	0.15^1^	0.25 (0.07)	0.13 (0.04)
VAR_WInVisits_	1,598	8.83	2.74	0.09^1^	0.33 (0.07)	0.06 (0.04)
VAR_WIRT_	1,529	0.11	0.04	0.92^1^	0.22 (0.06)	0.00 (0.00)
VAR_WDRT_	1,533	0.12	0.05	1.08^1^	0.22 (0.07)	0.00 (0.00)
***Feeding***						
VAR_FI_	2,337	0.50	0.10	0.67^1^	0.08 (0.04)	0.02 (0.03)
VAR_FIDUR_	2,337	13.35	3.79	0.06^1^	0.23 (0.05)	0.02 (0.03)
VAR_FInVisits_	2,292	6.12	2.46	0.14^1^	0.47 (0.06)	0.08 (0.03)
VAR_FIRT_	2,292	0.058	0.006	2.50^1^	0.00 (0.00)	0.04 (0.03)
***Drinking***						
OFF_WI_	1,308	0.12	0.08	0.67	0.27 (0.07)	0.05 (0.04)
OFF_WD_	1,315	0.11	0.09	0.81	0.21 (0.07)	0.01 (0.04)
OFF_WIDUR_	1,517	0.11	0.06	0.55	0.15 (0.06)	0.10 (0.04)
OFF_WInVisits_	1,595	0.12	0.06	0.50	0.31 (0.07)	0.01 (0.03)
OFF_WIRT_	1,515	0.11	0.07	0.64	0.17 (0.06)	0.00 (0.00)
OFF_WDRT_	1,483	0.11	0.08	0.73	0.16 (0.06)	0.00 (0.00)
***Feeding***						
OFF_FI_	2,337	0.04	0.05	1.25	0.10 (0.04)	0.05 (0.03)
OFF_FIDUR_	2,337	0.04	0.04	1.00	0.26 (0.05)	0.01 (0.02)
OFF_FInVisits_	2,292	0.05	0.07	1.40	0.13 (0.04)	0.04 (0.03)
OFF_FIRT_	2,292	0.05	0.13	2.60	0.13 (0.05)	0.01 (0.03)

*CV*: coefficient of variation; *ADWI*: average daily water disappearance; *ADWD*: average daily water dispensed; *WIDUR*: average daily water intake duration; *WInVisits*: average number of daily water intake visits; *WIRT*: water disappearance rate; *WDRT*: water dispensed rate; *ADFI*: average daily feed intake; *FIDUR*: average daily feed intake duration; *FInVisits*: average daily feed intake visits; *FIRT*: feed intake rate; *VAR*_*WI*_, *VAR*_*WD*_, *VAR*_*WIDUR*_, *VAR*_*WInVisits*_, *VAR*_*WIRT*_, *VAR*_*WDRT*_, *VAR*_*FI*_, *VAR*_*FIDUR*_, *VAR*_*FInVisits*_, and *VAR*_*FIRT*_ are the day-to-day variation for corresponding drinking and feeding traits; *OFF*_*WI*_, *OFF*_*WD*_, *OFF*_*WIDUR*_, *OFF*_*WInVisits*_, *OFF*_*WIRT*_, *OFF*_*WDRT*_, *OFF*_*FI*_, *OFF*_*FIDUR*_, *OFF*_*FInVisits*_, and *OFF*_*FIRT*_ are the proportion of off days for corresponding drinking and feeding traits

^1^ Coefficients of day-to-day variation, calculated as the SD of VAR of the trait divided by the mean of the corresponding trait, e.g. $\sqrt{{\mathrm{VAR}}_{FI}}/\mathrm{ADFI}$;

Using only data on days for which a pig had both feeding and drinking data, 54% of pigs had at least one off-feed day, 96% of pigs had at least one off-water day, and 41% of pigs received at least one treatment. On a daily basis, 2% of days were classified as off-feed, 12% as off-water, and 1% as receiving a treatment. Results for the overlap between off-days and treatment are in Table 2. Co-incidences of off-feed and off-water days and treatment days were all significantly (*P* < 0.0001) greater than expected based on a Chi-square test, by a factor that ranged from 1.4 to 12.2 (Table 2), both on a daily basis and on a 7-day rolling window basis. On the basis of a 7-day rolling window, 10%, 41%, and 6% of windows had at least one off-feed, off-water, or treatment day, respectively.

**Table 2:** Percentages of observations with off-feed, off-water, and treatment events (on diagonal) and of observed (upper diagonal) and expected (with independence, below diagonal) concurrent off-feed and off-water days and days pigs received treatment on a daily and a 7-day window basis

On a daily basis	Off-feed	Off-water	Treatment
Off-feed	2.48	0.96	0.24
Off-water	0.24	12.50	0.29
Treatment	0.02	0.12	0.88
On a 7-day window basis			
Off-feed	9.72	5.82	2.67
Off-water	4.09	41.10	3.71
Treatment	0.60	2.45	**5.93**

All observed percentages of concurrent events (above diagonal) were greater than expected (below diagonal) based on a Chi-square test at *P* < 0.0001

### Heritabilities of feeding and drinking traits

Table 1 shows estimates of heritability and litter effects for the feeding and drinking traits. Drinking traits in general had moderate to high estimates of heritability, especially average daily water dispensed, duration, and number of visits (0.44 to 0.58). Similar estimates were observed for corresponding feeding traits (0.35 to 0.51). Note that water dispensed (ADWD) and dispensed rate (WDRT) had higher estimates of heritability than water disappearance (ADWI) and disappearance rate (WIRT), respectively. Interestingly, estimates of heritability increased for all drinking traits (to 0.42 to 0.60) when the corresponding feeding trait was fitted as a covariate. Similar increases were observed for feed intake and duration (to 0.42 and 0.55, respectively) when the corresponding drinking trait was fitted as a covariate. Day-to-day variation in drinking traits had moderate estimates of heritability (0.22 to 0.38), while corresponding feeding traits had a wider range of estimates (0 to 0.47). Day-to-day variation in the number of feed intake visits had the highest estimate of heritability (0.47), while day-to-day variation in feed intake and intake rate had low estimates of heritability (0.08 and 0, respectively). The proportion of off-days had low to moderate estimates of heritability for both drinking and feed intake traits (0.15 to 0.31 and 0.10 to 0.26, respectively). Litter effects were low for all traits, ranging from 0 to 0.13.

### Correlations among drinking and feeding traits

Table 3 shows estimates of genetic and phenotypic correlations among drinking and feeding traits. Most of the genetic correlation estimates among drinking traits were moderate to high (0.30 to 0.92), except for water disappearance rate with water intake duration and number of visits (0.10 and − 0.07). Note that water dispensed was highly correlated with water disappearance, both genetically and phenotypically (0.84 and 0.79). In contrast, most genetic correlations among feeding traits were estimated to be low (0 to 0.11), except for feed intake rate with average daily feed intake (0.44). The majority of phenotypic correlations among drinking traits were also moderate to high (0.31 to 0.90), except for water intake duration with intake rate (0.09) and number of water intake visits with dispense rate and intake rate (0.09 and − 0.08, respectively). Phenotypic correlations among feeding traits were low, except for feed intake rate with average daily feed intake and duration (0.51 and − 0.70, respectively).

**Table 3:** Estimates of genetic (upper triangle) and phenotypic (lower triangle) correlations (SE in parentheses) among drinking and among feeding traits in the finisher

Drinking traits						
	ADWI	ADWD	WIDUR	WInVisits	WIRT	WDRT
ADWI		0.84 (0.05)	0.68 (0.08)	0.47 (0.11)	0.69 (0.09)	0.82 (0.07)
ADWD	0.79 (0.01)		0.82 (0.06)	0.57 (0.09)	0.33 (0.14)	0.92 (0.03)
WIDUR	0.60 (0.02)	0.61 (0.02)		0.82 (0.04)	0.10 (0.15)	0.59 (0.09)
WInVisits	0.36 (0.03)	0.34 (0.03)	0.80 (0.01)		− 0.07 (0.13)	0.30 (0.13)
WIRT	0.78 (0.01)	0.53 (0.02)	0.09 (0.03)	− 0.08 (0.03)		0.44 (0.12)
WDRT	0.66 (0.02)	0.90 (0.01)	0.31 (0.03)	0.09 (0.03)	0.66 (0.02)	
Feeding traits						
	ADFI		FIDUR	FInVisits	FIRT	
ADFI			0.10 (0.10)	0.11 (0.10)	0.44 (0.08)	
FIDUR	0.15 (0.03)			0.08 (0.09)	0.00 (0.00)	
FInVisits	− 0.01 (0.03)		0.11 (0.03)		0.00 (0.00)	
FIRT	0.51 (0.02)		− 0.70 (0.01)	− 0.10 (0.02)		

*ADWI*: average daily water disappearance; *ADWD*: average daily water dispensed; *WIDUR*: average daily water intake duration; *WInVisits*: average number of daily water intake visits; *WIRT*: water disappearance rate; *WDRT*: water dispensed rate; *ADFI*: average daily feed intake; *FIDUR*: average daily feed intake duration; *FInVisits*: average number of daily feed intake visits; *FIRT*: feed intake rate

Table 4 shows estimates of phenotypic and genetic correlations between corresponding drinking and feeding traits. Surprisingly, the drinking traits were not highly correlated with feeding traits, either genetically (0.08 to 0.36) or phenotypically (0.13 to 0.39). The same was true for day-to-day variation and off-days traits.

**Table 4:** Estimates of phenotypic and genetic correlations (SE in parentheses) between corresponding drinking and feeding traits in the finisher

Drinking trait	Feeding trait	Genetic	Phenotypic
ADWI	ADFI	0.36 (0.10)	0.39 (0.03)
ADWD	ADFI	0.23 (0.10)	0.29 (0.03)
WIDUR	FIDUR	0.10 (0.09)	0.16 (0.03)
WInVisits	FInVisits	0.24 (0.08)	0.29 (0.03)
WIRT	FIRT	0.24 (0.12)	0.27 (0.03)
WDRT	FIRT	0.08 (0.11)	0.13 (0.03)
VAR_WI_	VAR_FI_	0.10 (0.23)	0.05 (0.03)
VAR_WD_	VAR_FI_	− 0.08 (0.23)	0.03 (0.03)
VAR_WIDUR_	VAR_FIDUR_	− 0.14 (0.14)	0.08 (0.03)
VAR_WInVisits_	VAR_FInVisits_	0.18 (0.10)	0.13 (0.03)
VAR_WIRT_	VAR_FIRT_	− 0.06 (1.58)	0.16 (0.05)
VAR_WDRT_	VAR_FIRT_	0.64 (2.08)	− 0.02 (0.03)
OFF_WI_	OFF_FI_	− 0.25 (0.20)	− 0.10 (0.03)
OFF_WD_	OFF_FI_	0.04 (0.24)	− 0.14 (0.03)
OFF_WIDUR_	OFF_FIDUR_	− 0.29 (0.16)	− 0.05 (0.03)
OFF_WInVisits_	OFF_FInVisits_	0.08 (0.16)	− 0.01 (0.03)
OFF_WIRT_	OFF_FIRT_	− 0.23 (0.25)	− 0.12 (0.03)
OFF_WDRT_	OFF_FIRT_	− 0.07 (0.28)	− 0.09 (0.03)

*ADWI*: average daily water disappearance; *ADWD*: average daily water dispensed; *WIDUR*: average daily water intake duration; *WInVisit*s: average number of daily water intake visits; *WIRT*: water disappearance rate; *WDRT*: water dispensed rate; *ADFI*: average daily feed intake;* FIDUR*: average daily feed intake duration; *FInVisits*: average number of daily feed intake visits; *FIRT*: feed intake rate; *VAR*_*WI*_, *VAR*_*WD*_, *VAR*_*WIDUR*_, *VAR*_*WInVisits*_, *VAR*_*WIRT*_, *VAR*_*WDRT*_, *VAR*_*FI*_, *VAR*_*FIDUR*_, *VAR*_*FInVisits*_, and *VAR*_*FIRT*_ are the day-to-day variation for corresponding drinking and feeding traits; *OFF*_*WI*_, *OFF*_*WD*_, *OFF*_*WIDUR*_, *OFF*_*WInVisits*_, *OFF*_*WIRT*_, *OFF*_*WDRT*_, *OFF*_*FI*_, *OFF*_*FIDUR*_, *OFF*_*FInVisits*_, and *OFF*_*FIRT*_ are the proportion of off days for corresponding drinking and feeding traits

### Correlations of drinking and feeding traits with growth traits

Estimates of genetic and phenotypic correlations of drinking and feeding traits with growth rate in the three phases (quarantine nursery, challenge nursery, and finisher) are shown in Table 5. Drinking traits in general had low positive genetic correlation estimates with growth rate in the quarantine nursery but moderate positive genetic correlation estimates with growth rate in the challenge nursey and finisher. This means that, genetically, pigs that had higher growth rate under challenge, drank more water in the finisher, spent more time drinking, paid more visits to the drinker, and drank faster. Average daily feed intake and feed intake rate had moderate-to-high positive genetic correlation estimates with growth rate in the challenge nursey and finisher but lower genetic correlations with growth rate in quarantine nursery. Feed intake duration and number of visits, however, had very low and even negative genetic correlation estimates with growth rate in the different phases. This indicates that, genetically, pigs that had a higher growth rate under challenge, ate more feed and ate faster in the finisher. Fitting feeding or drinking traits as covariates for the corresponding drinking and feeding traits, respectively, had minimal effects on the correlation estimates for growth rate, except for genetic correlations of ADWI and ADWD with finisher ADG, which decreased to near zero.

**Table 5:** Estimates of phenotypic and genetic correlations (SE in parentheses) of drinking and feeding traits in the finisher with growth rate (ADG) in three phases

Trait	Quarantine nursery		Challenge nursery		Finisher	
	Genetic	Phenotypic	Genetic	Phenotypic	Genetic	Phenotypic
***Drinking***						
ADWI	0.10 (0.13)	0.06 (0.03)	0.39 (0.13)	0.27 (0.03)	0.29 (0.12)	0.34 (0.03)
ADWD	0.13 (0.11)	0.06 (0.03)	0.22 (0.12)	0.19 (0.03)	0.22 (0.11)	0.25 (0.03)
WIDUR	0.15 (0.10)	0.02 (0.03)	0.17 (0.11)	0.10 (0.03)	0.23 (0.10)	0.27 (0.03)
WInVisits	0.04 (0.10)	− 0.03 (0.03)	0.07 (0.11)	− 0.02 (0.03)	0.17 (0.10)	0.18 (0.03)
WIRT	0.14 (0.14)	0.10 (0.03)	0.34 (0.14)	0.31 (0.03)	0.19 (0.14)	0.27 (0.03)
WDRT	0.13 (0.13)	0.08 (0.03)	0.15 (0.14)	0.22 (0.03)	0.13 (0.13)	0.21 (0.03)
ADWI|ADFI	− 0.04 (0.12)	− 0.02 (0.03)	0.24 (0.14)	0.15 (0.03)	− 0.02 (0.13)	0.01 (0.04)
ADWD|ADFI	0.04 (0.11)	− 0.01 (0.03)	0.04 (0.13)	0.10 (0.03)	0.00 (0.12)	− 0.01 (0.04)
WIDUR|FIDUR	0.12 (0.10)	0.02 (0.03)	0.15 (0.11)	0.13 (0.11)	0.23 (0.10)	0.25 (0.03)
WInVisits|FInVisits	0.06 (0.10)	− 0.02 (0.03)	0.10 (0.11)	0.01 (0.03)	0.13 (0.10)	0.19 (0.03)
***Feeding***						
ADFI	0.27 (0.10)	0.23 (0.02)	0.49 (0.09)	0.42 (0.02)	0.84 (0.03)	0.86 (0.01)
FIDUR	0.10 (0.09)	0.01 (0.03)	− 0.09 (0.11)	− 0.08 (0.03)	0.09 (0.10)	0.16 (0.03)
FInVisits	− 0.14 (0.08)	− 0.10 (0.03)	− 0.03 (0.10)	− 0.08 (0.03)	− 0.01 (0.09)	− 0.03 (0.03)
FIRT	0.08 (0.09)	0.15 (0.03)	0.34 (0.09)	0.40 (0.02)	0.35 (0.09)	0.42 (0.02)
ADFI|ADWI	0.27(0.12)	0.21 (0.03)	0.49 (0.11)	0.46 (0.03)	0.82 (0.04)	0.86 (0.01)
ADFI|ADWD	0.27 (0.11)	0.22 (0.03)	0.54 (0.10)	0.48 (0.02)	0.80 (0.04)	0.86 (0.01)
FIDUR|WIDUR	0.12 (0.10)	0.01 (0.03)	− 0.12 (0.12)	− 0.16 (0.03)	− 0.01 (0.11)	0.11 (0.03)
FInVisits|WInVisits	− 0.22 (0.10)	− 0.09 (0.03)	− 0.13 (0.12)	− 0.10 (0.03)	0.03 (0.11)	− 0.06 (0.03)
***Drinking***						
VAR_WI_	0.10 (0.13)	− 0.01 (0.03)	0.23 (0.15)	0.15 (0.03)	0.21 (0.13)	0.17 (0.03)
VAR_WD_	0.05 (0.13)	0.00 (0.03)	0.10 (0.14)	0.12 (0.03)	0.20 (0.13)	0.15 (0.03)
VAR_WIDUR_	0.09 (0.12)	0.01 (0.03)	0.03 (0.14)	0.08 (0.03)	0.27 (0.12)	0.12 (0.03)
VAR_WInVisits_	0.01 (0.12)	− 0.02 (0.03)	− 0.07 (0.14)	0.03 (0.03)	0.08 (0.13)	− 0.01 (0.03)
VAR_WIRT_	0.10 (0.15)	0.04 (0.03)	0.13 (0.16)	0.14 (0.03)	0.09 (0.16)	0.14 (0.03)
VAR_WDRT_	0.13 (0.15)	0.04 (0.03)	0.14 (0.17)	0.13 (0.03)	0.13 (0.15)	0.13 (0.03)
***Feeding***						
VAR_FI_	0.55 (0.20)	0.15 (0.02)	0.65 (0.27)	0.17 (0.02)	0.30 (0.22)	− 0.04 (0.02)
VAR_FIDUR_	0.11 (0.12)	0.03 (0.03)	− 0.31 (0.13)	− 0.18 (0.02)	− 0.23 (0.12)	− 0.31 (0.02)
VAR_FInVisits_	− 0.11 (0.09)	− 0.06 (0.03)	− 0.20 (0.11)	− 0.09 (0.02)	0.01 (0.10)	−0.12 (0.02)
VAR_FIRT_	0.40 (0.87)	0.00 (0.02)	− 0.37 (1.25)	− 0.03 (0.02)	0.16 (0.86)	− 0.08 (0.02)
***Drinking***						
OFF_WI_	0.03 (0.14)	0.05 (0.03)	0.38 (0.15)	0.19 (0.03)	0.19 (0.14)	0.16 (0.03)
OFF_WD_	0.11 (0.15)	0.03 (0.03)	0.01 (0.18)	0.15 (0.03)	− 0.05 (0.17)	0.16 (0.03)
OFF_WIDUR_	0.18 (0.14)	0.03 (0.03)	0.12 (0.16)	0.06 (0.03)	0.11 (0.15)	0.04 (0.03)
OFF_WInVisits_	0.10 (0.13)	0.00 (0.03)	− 0.03 (0.14)	0.01 (0.03)	− 0.04 (0.13)	− 0.06 (0.03)
OFF_WIRT_	− 0.02 (0.18)	0.04 (0.03)	0.31 (0.19)	0.19 (0.03)	0.00 (0.19)	0.10 (0.03)
OFF_WDRT_	0.24 (0.18)	0.07 (0.03)	0.24 (0.21)	0.16 (0.03)	0.18 (0.18)	0.14 (0.03)
***Feeding***						
OFF_FI_	− 0.15 (0.17)	− 0.07 (0.02)	− 0.35 (0.17)	− 0.21 (0.02)	− 0.80 (0.08)	− 0.71 (0.01)
OFF_FIDUR_	0.02 (0.13)	0.03 (0.03)	0.03 (0.14)	− 0.02 (0.03)	− 0.51 (0.10)	− 0.46 (0.02)
OFF_FInVisits_	0.36 (0.13)	0.12 (0.02)	0.08 (0.16)	0.10 (0.03)	− 0.01 (0.15)	− 0.07 (0.02)
OFF_FIRT_	0.03 (0.15)	− 0.03 (0.02)	− 0.39 (0.16)	− 0.24 (0.02)	− 0.42 (0.13)	− 0.47 (0.02)

*ADWI*: average daily water disappearance; *ADWD*: average daily water dispensed; *WIDUR*: average daily water intake duration; *WInVisits*: average number of daily water intake visits; *WIRT*: water disappearance rate; *WDRT*: water dispensed rate; *ADFI*: average daily feed intake; *FIDUR*: average daily feed intake duration; *FInVisits*: average number of daily feed intake visits; *FIRT*: feed intake rate; *ADWI|ADFI*, *ADWD|ADFI*, *WIDUR|FIDUR*, *WInVisits|FInVisits*, *ADFI|ADWI*, *ADFI|ADWD*, *FIDUR|WIDUR*, *FInVisits|WInVisits* are the fitting the second trait as covariate for the first trait; *VAR*_*WI*_, *VAR*_*WD*_, *VAR*_*WIDUR*_, *VAR*_*WInVisits*_, *VAR*_*WIRT*_, *VAR*_*WDRT*_, *VAR*_*FI*_, *VAR*_*FIDUR*_, *VAR*_*FInVisits*_, and *VAR*_*FIRT*_ are the day-to-day variation for corresponding drinking and feeding traits; *OFF*_*WI*_, *OFF*_*WD*_, *OFF*_*WIDUR*_, *OFF*_*WInVisits*_, *OFF*_*WIRT*_, *OFF*_*WDRT*_, *OFF*_*FI*_, *OFF*_*FIDUR*_, *OFF*_*FInVisits*_, and *OFF*_*FIRT*_ are the proportion of off days for corresponding drinking and feeding traits

Most day-to-day variation traits for drinking traits had low positive genetic correlation estimates with growth rate in the quarantine and challenge nursery. Estimates of genetic correlations with growth rate in finisher were stronger for day-to-day variation in water disappearance, dispensed, and duration (0.21, 0.20, and 0.27, respectively) than for day-to-day variation in number of visits, disappearance rate, and dispensed rate. Day-to-day variation in feeding traits had stronger genetic correlation estimates with growth rate than day-to-day variation in the corresponding drinking traits. Day-to-day variation in daily feed intake had high positive genetic correlation estimates with growth rate in the quarantine and challenge nursery (0.55 and 0.65) and a moderate genetic correlation estimate with growth rate in the finisher (0.30). Day-to-day variation in feed intake duration and number of visits, however, had moderate negative genetic correlation estimates with growth rate in the challenge nursery and finisher. Day-to-day variation in feed intake rate had very low estimates of heritability and, thus, had genetic correlation estimates with high standard errors. For the proportion of off-water or -feed days, off-water traits generally had lower genetic correlation estimates with growth rate than off-feed traits. Off-days for feed intake and intake rate had moderate-to-high negative genetic correlation estimates with growth rate in the challenge nursery and finisher (− 0.35 to − 0.80). Off-days for feed intake duration also had a high negative genetic correlation estimate with growth rate in the finisher. Estimates of phenotypic correlations in general were of a similar magnitude as genetic correlation estimates for off-days for all drinking and feeding traits.

### Correlations of drinking and feeding traits with clinical disease traits

Table 6 shows estimates of phenotypic correlations of drinking and feeding traits with health scores and with treatment rate in the different phases. Phenotypic correlations with mortality rates were not computed because only pigs that survived were included for the drinking and feeding trait analyses. Drinking traits generally had low phenotypic correlation estimates with health scores and treatment rates. Average daily feed intake and intake rate, however, had moderately high phenotypic correlation estimates with health score in the finisher (0.39 and 0.24) and with treatment rates (− 0.18 to − 0.30 for feed intake and − 0.09 to − 0.17 for intake rate). Fitting feeding or drinking traits as covariates for the corresponding drinking and feeding traits, respectively, did not substantially change their correlation estimates with health scores and treatment rates, except for ADWI and ADWD, for which phenotypic correlations decreased. Similar trends were found for day-to-day variation and off-days for drinking traits, which generally had low phenotypic correlation estimates with health scores and treatment rates. By contrast, day-to-day variation and off-days for feed intake and duration traits tended to have stronger phenotypic correlation estimates with health score in the finisher and with treatment rates. The proportion of off-feed days for intake rate also had strong phenotypic correlation estimates with health scores and treatment rates.

**Table 6:** Estimates of phenotypic correlations (SE in parentheses) of drinking and feeding traits in the finisher with health scores and treatment rates in different phases

Trait	Health score		Treatment rate		
	cNursery	Finisher	cNursery	Finisher	Combined
***Drinking***					
ADWI	0.11 (0.03)	0.19 (0.03)	− 0.13 (0.03)	− 0.03 (0.03)	− 0.12 (0.03)
ADWD	0.03 (0.03)	0.13 (0.03)	− 0.11 (0.03)	− 0.02 (0.03)	− 0.09 (0.03)
WIDUR	0.00 (0.03)	0.03 (0.03)	− 0.11 (0.03)	− 0.02 (0.03)	− 0.09 (0.03)
WInVisits	− 0.04 (0.03)	− 0.04 (0.03)	− 0.10 (0.03)	− 0.04 (0.03)	− 0.10 (0.03)
WIRT	0.16 (0.03)	0.26 (0.03)	− 0.13 (0.03)	− 0.07 (0.03)	− 0.13 (0.03)
WDRT	0.06 (0.03)	0.18 (0.03)	− 0.12 (0.03)	− 0.04 (0.03)	− 0.11 (0.03)
ADWI|ADFI	0.05 (0.03)	0.03 (0.03)	− 0.06 (0.03)	0.04 (0.03)	− 0.01 (0.03)
ADWD|ADFI	− 0.01 (0.03)	0.02 (0.03)	− 0.06 (0.03)	0.03 (0.03)	− 0.02 (0.03)
WIDUR|FIDUR	0.01 (0.03)	0.03 (0.03)	− 0.12 (0.03)	− 0.02 (0.03)	− 0.09 (0.03)
WInVisits|FInVisits	− 0.05 (0.03)	− 0.03 (0.03)	− 0.10 (0.03)	− 0.04 (0.03)	− 0.09 (0.03)
***Feeding***					
ADFI	0.14 (0.02)	0.39 (0.02)	− 0.27 (0.02)	− 0.18 (0.02)	− 0.30 (0.02)
FIDUR	− 0.03 (0.02)	0.02 (0.02)	0.00 (0.02)	− 0.03 (0.02)	− 0.01 (0.02)
FInVisits	− 0.03 (0.02)	− 0.05 (0.02)	− 0.02 (0.02)	− 0.01 (0.02)	− 0.03 (0.02)
FIRT	0.12 (0.02)	0.24 (0.02)	− 0.17 (0.02)	− 0.09 (0.02)	− 0.17 (0.02)
ADFI|ADWI	0.15 (0.03)	0.37 (0.03)	− 0.22 (0.03)	− 0.17 (0.13)	− 0.25 (0.03)
ADFI|ADWD	0.17 (0.03)	0.38 (0.03)	− 0.24 (0.03)	− 0.16 (0.03)	− 0.25 (0.03)
FIDUR|WIDUR	− 0.06 (0.03)	0.00 (0.03)	0.03 (0.03)	− 0.01 (0.03)	0.01 (0.03)
FInVisits|WInVisits	0.01 (0.03)	− 0.03 (0.03)	0.01 (0.03)	− 0.01 (0.03)	− 0.01 (0.03)
***Drinking***					
VAR_WI_	0.04 (0.03)	0.12 (0.03)	− 0.06 (0.03)	0.00 (0.03)	− 0.04 (0.03)
VAR_WD_	0.01 (0.03)	0.09 (0.03)	− 0.07 (0.03)	0.02 (0.03)	− 0.04 (0.03)
VAR_WIDUR_	0.03 (0.03)	0.04 (0.03)	− 0.06 (0.03)	0.03 (0.03)	− 0.02 (0.03)
VAR_WInVisits_	− 0.01 (0.03)	− 0.04 (0.03)	0.03 (0.03)	0.06 (0.03)	0.05 (0.03)
VAR_WIRT_	0.06 (0.03)	0.19 (0.03)	− 0.08 (0.03)	− 0.04 (0.03)	− 0.09 (0.03)
VAR_WDRT_	0.04 (0.03)	0.15 (0.03)	− 0.07 (0.03)	− 0.04 (0.03)	− 0.08 (0.03)
***Feeding***					
VAR_FI_	0.10 (0.02)	0.09 (0.02)	0.18 (0.02)	0.03 (0.02)	0.15 (0.02)
VAR_FIDUR_	− 0.03 (0.02)	− 0.21 (0.02)	0.23 (0.02)	0.19 (0.02)	0.27 (0.02)
VAR_FInVisits_	− 0.03 (0.03)	− 0.11 (0.02)	0.06 (0.02)	0.09 (0.02)	0.07 (0.02)
VAR_FIRT_	− 0.01 (0.02)	− 0.06 (0.02)	0.01 (0.02)	0.05 (0.02)	0.04 (0.02)
***Drinking***					
OFF_WI_	0.05 (0.03)	0.14 (0.03)	0.00 (0.03)	0.03 (0.03)	0.01 (0.03)
OFF_WD_	0.05 (0.03)	0.10 (0.03)	− 0.07 (0.03)	− 0.04 (0.03)	− 0.06 (0.03)
OFF_WIDUR_	0.00 (0.03)	− 0.03 (0.03)	− 0.04 (0.03)	0.06 (0.03)	0.01 (0.03)
OFF_WInVisits_	0.00 (0.03)	− 0.06 (0.03)	0.07 (0.03)	0.07 (0.03)	0.08 (0.03)
OFF_WIRT_	0.08 (0.03)	0.20 (0.03)	− 0.05 (0.03)	− 0.01 (0.03)	− 0.05 (0.03)
OFF_WDRT_	0.06 (0.03)	0.15 (0.03)	− 0.08 (0.03)	− 0.03 (0.03)	− 0.08 (0.03)
***Feeding***					
OFF_FI_	− 0.07 (0.02)	− 0.35 (0.02)	0.31 (0.02)	0.25 (0.02)	0.38 (0.02)
OFF_FIDUR_	0.03 (0.02)	− 0.16 (0.02)	0.21 (0.02)	0.17 (0.02)	0.24 (0.02)
OFF_FInVisits_	0.03 (0.02)	− 0.03 (0.02)	0.10 (0.02)	0.10 (0.02)	0.12 (0.02)
OFF_FIRT_	− 0.10 (0.02)	− 0.36 (0.02)	0.24 (0.02)	0.16 (0.02)	0.24 (0.02)

*cNursery*: challenge nursery; *Combined*: challenge nursery and finisher combined; *ADWI*: average daily water disappearance; *ADWD*: average daily water dispensed; *WIDUR*: average daily water intake duration; *WInVisits*: average number of daily water intake visits; *WIRT*: water disappearance rate; *WDRT*: water dispensed rate;* ADFI*: average daily feed intake; *FIDUR*: average daily feed intake duration; *FInVisits*: average number of daily feed intake visits; *FIRT*: feed intake rate; *ADWI|ADFI*, *ADWD|ADFI*, *WIDUR|FIDUR*, *WInVisits|FInVisits*, *ADFI|ADWI*, *ADFI|ADWD*, *FIDUR|WIDUR*, *FInVisits|WInVisits* are the fitting the second trait as covariate for the first trait; *VAR*_*WI*_, *VAR*_*WD*_, *VAR*_*WIDUR*_, *VAR*_*WInVisits*_, *VAR*_*WIRT*_, *VAR*_*WDRT*_, *VAR*_*FI*_, *VAR*_*FIDUR*_, *VAR*_*FInVisits*_, and *VAR*_*FIRT*_ are the day-to-day variation for corresponding drinking and feeding traits; *OFF*_*WI*_, *OFF*_*WD*_, *OFF*_*WIDUR*_, *OFF*_*WInVisits*_, *OFF*_*WIRT*_, *OFF*_*WDRT*_, *OFF*_*FI*_, *OFF*_*FIDUR*_, *OFF*_*FInVisits*_, and *OFF*_*FIRT*_ are the proportion of off days for corresponding drinking and feeding traits

Estimates of genetic correlations of drinking and feeding traits with clinical disease traits are shown in Table 7. Treatment rate in the finisher had a zero estimate of heritability and was, thus, excluded from these analyses. Interestingly, water intake duration and number of visits had relatively stronger negative genetic correlation estimates with treatment and mortality rates than other traits, especially across the challenge nursery and finisher (− 0.39 and − 0.45 for treatment rate; − 0.20 and − 0.19 for mortality). This implies that, genetically, pigs that were more likely to die or that received more treatments, spent less time drinking and paid fewer visits to drinker. Average daily water disappearance and dispensed were genetically positively correlated with health scores and negatively correlated with treatment and mortality rates, but these correlation estimates were not strong in general. Interestingly, water disappearance rate had moderate genetic correlation estimates with health scores in the challenge nursery and finisher (0.41 and 0.36), which means that pigs with higher health scores, genetically had a higher water disappearance rate. For feeding traits, feed intake duration and number of visits had moderate negative genetic correlation estimates with mortality in the finisher and across the challenge nursery and finisher (− 0.23 to − 0.43), which means that, genetically, pigs that were more likely to die spent less time eating and paid fewer visits to feeder. Average daily feed intake had moderate genetic correlation estimates with treatment rate (− 0.25 to − 0.33) and health score in the finisher (0.31). Fitting feeding or drinking traits as covariates for the corresponding drinking and feeding traits, respectively, in general had minimal effect on estimates of genetic correlations with clinical traits, except for ADWI and ADWD with health score in the finisher and with treatment rate across the challenge nursery and finisher, for which adding the covariate decreased genetic correlation estimates.

**Table 7:** Estimates of genetic correlations (SE in parentheses) of drinking and feeding traits in the finisher with health scores, treatment rates, and mortality in different phases

Trait	Health score	Treatment rate		Mortality			
	cNursery	Finisher	cNursery	Combined	cNursery	Finisher	Combined
***Drinking***							
ADWI	0.09 (0.21)	0.30 (0.20)	− 0.13 (0.18)	− 0.18 (0.24)	− 0.18 (0.19)	− 0.10 (0.23)	− 0.15 (0.17)
ADWD	− 0.01 (0.19)	0.22 (0.19)	− 0.20 (0.16)	− 0.24 (0.21)	− 0.06 (0.17)	− 0.29 (0.20)	− 0.20 (0.14)
WIDUR	− 0.21 (0.18)	0.10 (0.17)	− 0.27 (0.14)	− 0.39 (0.19)	− 0.10 (0.15)	− 0.18 (0.18)	− 0.20 (0.13)
WInVisits	− 0.14 (0.17)	− 0.18 (0.17)	− 0.33 (0.14)	− 0.45 (0.19)	− 0.24 (0.16)	− 0.02 (0.17)	− 0.19 (0.13)
WIRT	0.41 (0.20)	0.36 (0.20)	− 0.05 (0.20)	0.04 (0.25)	0.02 (0.22)	− 0.01 (0.23)	0.12 (0.18)
WDRT	0.15 (0.19)	0.24 (0.20)	− 0.03 (0.18)	− 0.08 (0.23)	0.07 (0.20)	− 0.20 (0.21)	− 0.04 (0.16)
ADWI|ADFI	− 0.01 (0.14)	0.11 (0.21)	− 0.19 (0.16)	− 0.01 (0.23)	− 0.23 (0.18)	− 0.10 (0.20)	− 0.16 (0.14)
ADWD|ADFI	− 0.08 (0.13)	0.08 (0.19)	− 0.16 (0.15)	− 0.13 (0.21)	− 0.10 (0.16)	− 0.25 (0.19)	− 0.11 (0.15)
WIDUR|FIDUR	− 0.13 (0.12)	0.10 (0.17)	− 0.26 (0.13)	− 0.37 (0.19)	− 0.26 (0.15)	− 0.17 (0.18)	− 0.20 (0.13)
WInVisits|FInVisits	− 0.13 (0.11)	− 0.17 (0.17)	− 0.34 (0.13)	− 0.48 (0.18)	− 0.31 (0.15)	0.02 (0.17)	− 0.16 (0.12)
***Feeding***							
ADFI	0.02 (0.16)	0.31 (0.15)	− 0.25 (0.14)	− 0.33 (0.17)	0.02 (0.15)	− 0.12 (0.18)	− 0.13 (0.20)
FIDUR	− 0.11 (0.16)	0.05 (0.16)	− 0.03 (0.14)	0.00 (0.20)	0.40 (0.15)	− 0.32 (0.16)	− 0.43 (0.18)
FInVisits	− 0.01 (0.15)	− 0.13 (0.15)	− 0.05 (0.13)	0.06 (0.19)	− 0.10 (0.14)	− 0.27 (0.16)	− 0.23 (0.11)
FIRT	0.00 (0.14)	0.10 (0.15)	− 0.06 (0.14)	− 0.12 (0.17)	− 0.32 (0.15)	− 0.02 (0.15)	0.10 (0.17)
ADFI|ADWI	0.20 (0.13)	0.36 (0.17)	− 0.16 (0.15)	− 0.35 (0.19)	0.08 (0.18)	0.03 (0.20)	− 0.03 (0.15)
ADFI|ADWD	0.23 (0.13)	0.37 (0.16)	− 0.18 (0.15)	− 0.32 (0.19)	0.03 (0.17)	− 0.02 (0.19)	− 0.06 (0.15)
FIDUR|WIDUR	0.10 (0.12)	0.01 (0.18)	0.02 (0.14)	− 0.01 (0.20)	0.40 (0.16)	− 0.08 (0.18)	0.07 (0.13)
FInVisits|WInVisits	− 0.13 (0.13)	− 0.01 (0.19)	0.20 (0.14)	0.28 (0.21)	0.00 (0.16)	− 0.21 (0.19)	− 0.15 (0.14)
***Drinking***							
VAR_WI_	0.09 (0.22)	0.16 (0.22)	− 0.10 (0.19)	− 0.15 (0.27)	0.00 (0.20)	− 0.10 (0.22)	− 0.06 (0.17)
VAR_WD_	− 0.16 (0.21)	0.04 (0.21)	− 0.19 (0.18)	− 0.14 (0.23)	0.10 (0.19)	− 0.33 (0.21)	− 0.17 (0.16)
VAR_WIDUR_	− 0.22 (0.22)	0.28 (0.20)	− 0.23 (0.17)	− 0.43 (0.26)	0.26 (0.19)	− 0.16 (0.25)	0.01 (0.15)
VAR_WInVisits_	− 0.16 (0.21)	− 0.14 (0.21)	− 0.15 (0.18)	− 0.28 (0.24)	0.17 (0.18)	0.18 (0.22)	0.13 (0.15)
VAR_WIRT_	0.21 (0.23)	0.27 (0.23)	− 0.11 (0.21)	− 0.05 (0.30)	− 0.01 (0.23)	0.09 (0.24)	0.10 (0.20)
VAR_WDRT_	0.12 (0.23)	0.07 (0.24)	0.00 (0.21)	− 0.01 (0.27)	0.21 (0.23)	− 0.02 (0.24)	0.14 (0.20)
***Feeding***							
VAR_FI_	0.22 (0.29)	− 0.23 (0.31)	0.14 (0.25)	− 0.30 (0.38)	0.71 (0.29)	0.45 (0.36)	0.56 (0.35)
VAR_FIDUR_	− 0.21 (0.20)	− 0.32 (0.19)	0.11 (0.17)	0.03 (0.25)	0.65 (0.19)	− 0.17 (0.20)	− 0.24 (0.23)
VAR_FInVisits_	− 0.19 (0.12)	− 0.30 (0.15)	0.07 (0.12)	0.09 (0.19)	− 0.01 (0.15)	0.04 (0.17)	0.05 (0.11)
VAR_FIRT_	− 0.73 (1.74)	− 0.20 (0.87)	0.35 (1.15)	0.85 (2.85)	− 0.82 (1.48)	NA	NA
***Drinking***							
OFF_WI_	− 0.11 (0.22)	0.20 (0.22)	− 0.04 (0.20)	− 0.18 (0.25)	− 0.29 (0.21)	− 0.04 (0.24)	− 0.16 (0.18)
OFF_WD_	− 0.34 (0.23)	− 0.14 (0.24)	− 0.25 (0.20)	− 0.10 (0.29)	− 0.21 (0.23)	− 0.46 (0.26)	− 0.37 (0.18)
OFF_WIDUR_	− 0.18 (0.19)	0.06 (0.23)	− 0.19 (0.20)	− 0.46 (0.32)	0.27 (0.22)	− 0.18 (0.27)	0.04 (0.18)
OFF_WInVisits_	− 0.07 (0.17)	− 0.27 (0.19)	− 0.02 (0.18)	− 0.12 (0.23)	0.31 (0.19)	0.10 (0.20)	0.22 (0.16)
OFF_WIRT_	0.38 (0.27)	0.23 (0.26)	− 0.16 (0.24)	− 0.08 (0.34)	− 0.08 (0.27)	0.18 (0.28)	0.10 (0.23)
OFF_WDRT_	0.31 (0.28)	0.18 (0.29)	−0.24 (0.25)	− 0.30 (0.30)	− 0.10 (0.29)	− 0.13 (0.30)	− 0.12 (0.24)
***Feeding***							
OFF_FI_	− 0.06 (0.24)	− 0.45 (0.21)	0.38 (0.19)	0.51 (0.23)	0.27 (0.24)	0.28 (0.30)	0.48 (0.25)
OFF_FIDUR_	0.18 (0.19)	− 0.16 (0.19)	0.26 (0.17)	0.08 (0.21)	− 0.15 (0.19)	0.26 (0.20)	0.24 (0.20)
OFF_FInVisits_	− 0.02 (0.18)	− 0.03 (0.22)	0.20 (0.18)	0.14 (0.25)	0.23 (0.24)	0.32 (0.23)	0.34 (0.17)
OFF_FIRT_	0.01 (0.20)	− 0.36 (0.23)	0.28 (0.19)	0.48 (0.24)	0.60 (0.27)	− 0.09 (0.27)	NA

*cNursery*: challenge nursery; *Combined*: challenge nursery and finisher combined; *ADWI*: average daily water disappearance; *ADWD*: average daily water dispensed; *WIDUR*: average daily water intake duration; *WInVisits*: average number of daily water intake visits; *WIRT*: water disappearance rate; *WDRT*: water dispensed rate; *ADFI*: average daily feed intake; *FIDUR*: average daily feed intake duration;* FInVisits*: average number of daily feed intake visits;* FIRT*: feed intake rate; *ADWI|ADFI*, *ADWD|ADFI*, *WIDUR|FIDUR*, *WInVisits|FInVisits*, *ADFI|ADWI*, *ADFI|ADWD*, *FIDUR|WIDUR*, *FInVisits|WInVisits* are the fitting the second trait as covariate for the first trait; *VAR*_*WI*_, *VAR*_*WD*_, *VAR*_*WIDUR*_, *VAR*_*WInVisits*_, *VAR*_*WIRT*_, *VAR*_*WDRT*_, *VAR*_*FI*_, *VAR*_*FIDUR*_, *VAR*_*FInVisits*_, and *VAR*_*FIRT*_ are the day-to-day variation for corresponding drinking and feeding traits; *OFF*_*WI*_, *OFF*_*WD*_, *OFF*_*WIDUR*_, *OFF*_*WInVisits*_, *OFF*_*WIRT*_, *OFF*_*WDRT*_, *OFF*_*FI*_, *OFF*_*FIDUR*_, *OFF*_*FInVisits*_, and *OFF*_*FIRT*_ are the proportion of off days for corresponding drinking and feeding traits

Day-to-day variation in drinking traits in general had low to moderate genetic correlations with health scores and with treatment and mortality rates (Table 7). However, these correlation estimates, e.g., of day-to-day variation in water intake duration with treatment rate (− 0.23 to − 0.43) had unexpected directions because pigs that are less resilient were expected to have higher day-to-day variation in drinking traits. Day-to-day variation in feeding traits generally had moderate to high genetic correlation estimates with resilience traits and in the expected direction.

Most off-day traits for drinking behavior had low genetic correlation estimates with health scores and with treatment and mortality rates, except for off-days for water dispensed with mortality in the finisher and across the nursery and finisher (− 0.46 and − 0.37) and for off-days for water intake duration with treatment rate across the nursery and finisher (− 0.46), but again in the unexpected direction. On the other hand, the proportion of off-days for for feed intake had strong genetic correlation estimates with health score in the finisher (− 0.45), with treatment rate in the finisher and across the nursery and finisher (0.38 and 0.51), and with mortality across the nursery and finisher (0.48), all in the expected direction. Off-days for number of feed intake visits had moderate positive genetic correlation estimates with mortality in the finisher and across the nursery and finisher (0.32 and 0.34). Off-feed days for feed intake rate had moderate to high genetic correlation estimates with health score in the finisher (− 0.36), with treatment rate in the finisher and across the nursery and finisher (0.28 and 0.48), and with mortality in the challenge nursery (0.60), all in the expected direction.

## Discussion

Although several studies have explored the impact of water intake on pig production performance and health [10–12], very few studies have addressed the effect of health status on individual drinking traits [15]. To our knowledge, this is the first study to report genetic parameters of individual drinking behaviors under a disease challenge. In this study, we were also able to combine feeding and drinking data and explore the relationships between feeding and drinking behaviors under a severe disease challenge and the relationship of these traits with disease resilience. Because all animals were exposed to disease, as the objective was to evaluate variation in performance and behavior under disease, data on a comparable control group of pigs that were not exposed to disease were not available.

Water intake is difficult to measure because pigs like to play with water, especially in hot weather, which makes individual water intake data noisier than individual feed intake data. To address this, we developed stringent methods for quality control and editing of the drinking data prior to analysis. Although this removed a large proportion of the data (9% of water intake days and 32% to 41% of pigs, depending on the trait), we believe these data processing steps were critical to ensure that only data from days without obvious recording errors were used for analysis. In fact, estimates of heritability for the drinking traits increased by 50% to 100% following quality control editing. We don’t expect this stringent editing to have biased estimates of relationships of feeding and drinking traits with resilience traits because most data errors are technical in nature and expected to be independent of the health status of the pig. The resulting data and analyses provide novel insights into the phenotypic and genetic relationships among feeding and drinking behavior traits under disease, as well as into their relationships with disease resilience. Although large standard errors were observed for most parameter estimates, especially those of genetic correlations, the novelty of the traits investigated in relation to published literature merits interpretation and discussion of the trends that the estimates provide. It is, however, acknowledged that further validation is required before strong conclusions can be drawn. In addition, because the water intake data was limited to pigs that survived to slaughter, estimates must be interpreted in that context. Water intake data on pigs that died were eliminated to avoid the possibility that drinking traits on such pigs are genetically different from corresponding traits in pigs that survived.

### Genetic parameters of drinking and feeding traits under a severe disease challenge

#### Heritability

Many estimates of genetic parameters of feeding behaviors in generally healthy pigs have been reported [21–25]. Reported estimates of heritability for feed intake duration per day, number of visits per day, and feed intake rate range from 0.31 to 0.46, from 0.29 to 0.43, and from 0.41 to 0.50, respectively. These estimates were in general slightly lower than the estimates reported in this study (Table 1), where pigs were under a severe disease challenge. To our knowledge, no studies have reported genetic parameters of drinking traits. The drinking traits had estimates of heritability that were of a similar magnitude as those for feeding traits. Water intake duration and number of visits per day were the most heritable among the drinking traits and were more heritable than the corresponding feeding traits. Additionally, both feed and water intake duration and number of visits per day were more heritable than daily feed intake and daily water disappearance or dispensed. It should be noted that daily water dispensed was more heritable than water disappearance. The coefficient of variation was also much higher for water dispensed than water disappearance (Table 1), which shows that water dispensed had higher variability. The same holds true for rates of water dispensed and disappearance. However, water dispensed and disappearance were strongly correlated with each other, both phenotypically (0.79) and genetically (0.84), which was as expected, as both traits are a combination of water consumption, wastage, and playing behavior.

Putz et al. [8] and Cheng et al. [9] reported low to moderate estimates of heritability for day-to-day variation and the proportion of off-days derived from feed intake and duration. Similar results were also reported here because they were based on mostly the same feeding data (Table 1). Day-to-day variation in the number of feed intake visits, which was not investigated in these previous studies, was found to be highly heritable. In general, both day-to-day variation and the proportion of off-days for drinking traits were low to moderately heritable.

#### Relationships between drinking and feeding traits

In general, estimates of phenotypic and genetic correlations among feeding traits were very low (Table 3). Estimates of the phenotypic (0.15) and genetic (0.10) correlation between daily feed intake and duration were much lower than the range of estimates reported in the literature (0.17 to 0.88 and 0.14 to 0.97, respectively) [21, 23–25]. Cheng et al. [9] suggested that the low correlations in these data were probably because feed intake and duration were differently affected by the disease challenge. The average number of daily feed intake visits also had low phenotypic (− 0.01) and genetic (0.11) correlations with daily feed intake and were also quite different than estimates reported in the literature (− 0.4 to − 0.09 and − 0.35 to 0.02, respectively) [21, 23–25]. The average number of daily feed intake visits also had low phenotypic and genetic correlations with daily feed intake duration (0.11 and 0.08, respectively) but these estimates were in the range of estimates reported in the literature [21, 23–25] (− 0.12 to 0.15 and − 0.21 to 0.38, respectively). It is noteworthy that the range of correlation estimates in the literature is large, which could result from differences in feeding systems, breeds, environments, analytical models, health status, etc. The low correlations in our data may also because that the different feeding traits were differentially affected by disease challenge, as is evident from the large coefficients of day-to-day variation in feed intake, duration, number of visits, and intake rate (0.67, 0.06, 0.14, and 2.5, respectively, Table 1). Feed intake rate had moderate positive phenotypic (0.51) and genetic (0.44) correlation estimates with daily feed intake in our data (Table 3), as well as strong negative phenotypic (− 0.70) but very low genetic (0) correlations with duration, and very low phenotypic (0.10) and genetic (0) correlations with number of feed intake visits. Except for the low genetic correlation of feed intake rate with feed intake duration, these estimates were in general consistent with literature estimates (0.41 to 0.42 for phenotypic correlations and 0.20 to 0.49 for genetic correlations with daily feed intake; − 0.76 to − 0.70 for phenotypic correlations and − 0.86 to − 0.78 for genetic correlations with feed intake duration; − 0.15 to − 0.10 for phenotypic correlations and − 0.22 to − 0.12 for genetic correlations with number of feed intake visits) [21, 23]. Most estimates of phenotypic and genetic correlations among drinking traits were much higher than corresponding estimates among feeding traits (Table 3). Coefficients of day-to-day variation (Table 1) were more consistent across drinking than across feeding traits (0.09 to 1.08 versus 0.06 to 2.50, respectively, Table 1).

Although no studies have reported on genetic parameters of drinking traits, some studies have explored the phenotypic relationships among drinking traits. Using data from cameras, Kashiha et al. [26] found that water usage was highly related with the duration of drink nipple visits (R^2^ = 0.92). Maselyne et al. [27], using an radio frequency identification drinking system, found similar magnitudes of the relationships of water usage with the number of water intake visits (R^2^ = 0.69 to 0.75) and duration (R^2^ = 0.65 to 0.71). All these studies were, however, conducting using generally healthy pigs.

Phenotypic and genetic correlations between corresponding feeding and drinking traits were generally low (Table 4). This contradicts previous studies that suggest a high phenotypic relationship between feeding and drinking traits [15, 28]. However, these studies were conducted under conditions that were very different from our study, where pigs were under severe disease challenge, which may disproportionately affect feeding and drinking behavior, resulting in a reduction in the relationships between feeding and drinking traits. In the present study, pigs were severely challenged with multiple viral and bacterial pathogens, which resulted in chronic disease, particularly of the respiratory tract. Additionally, other factors, such as social stress and vaccination, could also affect drinking and feeding behaviors.

### Relationship of drinking and feeding traits with growth rate

Average daily feed intake was strongly correlated with average daily gain (ADG) in the finisher, both phenotypically (0.86) and genetically (0.84), which is consistent with the literature (0.67 to 0.73 for phenotypic correlations and 0.77 to 0.87 for for genetic correlations) [21, 24, 29], although pigs were under a severe disease challenge in this study. Feed intake rate was also moderately correlated with ADG in the finisher, both phenotypically (0.34) and genetically (0.42), and consistent with estimates in the same literature (0.25 to 0.28 for phenotypic correlations and 0.17 to 0.48 for genetic correlations) [21, 24, 29]. Interestingly, daily feed intake and intake rate (recorded in the finisher) were also moderately correlated with ADG in the challenge nursery, both phenotypically (0.40 to 0.42) and genetically (0.34 to 0.49), although ADG in the challenge nursey was weakly correlated with ADG in the finisher, both phenotypically (0.20) and genetically (0.15) [9]. This means that pigs that grew faster in the challenge nursery ate more feed and ate faster in the finisher. Feed intake duration and number of visits, however, had very low phenotypic (0.16 and − 0.03, respectively) and genetic (0.09 and − 0.01, respectively) correlations with ADG in the finisher but in the range of estimates reported in the literature (0.13 to 0.64 for phenotypic correlations and 0.02 to 0.69 for genetic correlations with duration; − 0.09 to 0.01 for phenotypic correlations and − 0.22 to − 0.03 for genetic correlations with number of visits) [21, 24, 29]. Drinking traits in general had low correlations with ADG in the nursery and the finisher, both phenotypically (− 0.02 to 0.34) and genetically (− 0.02 to 0.39). Very few studies have reported relationships of water intake with growth rate in pigs but Brew et al. [30] reported a very low phenotypic relationship (R^2^ = 0.005) of water intake with ADG in generally healthy growing beef cattle.

Day-to-day variation and the proportion of off-days for drinking traits had low phenotypic and genetic correlations with ADG in the nursery and the finisher (Table 5). In contrast, the proportion of off-days for feed intake, duration, and intake rate had moderate to strong negative phenotypic and genetic correlations with ADG in the finisher (− 0.42 to − 0.80), as was already established based on these data by Cheng et al. [9].

### Relationship of drinking and feeding traits with clinical disease traits

There was clear evidence that off-feed, off-water, and treatment events coincided more often than expected, by a factor of 1.4 to 12.2 (Table 2). This suggests that the recorded feeding and drinking phenotypes are related to the health status of pigs. However, feeding and drinking traits in general had low phenotypic correlations with clinical disease traits (Table 6), except for the proportion of off-feed intake days with health score in the finisher (− 0.35) and with treatment rate across the nursery and finisher (0.25 to 0.38). This could be because only 41% of pigs received at least one treatment during the finishing period and there were not many treatment days across all pigs. Putz et al. [8] and Cheng et al. [9] demonstrated that day-to-day variation and the proportion of off-days for feed intake and duration were highly associated with disease resilience, using the same feeding data as used herein. Drinking traits, however, consistently had low phenotypic correlations with clinical traits.

Estimates of genetic correlations of feeding and drinking traits with clinical traits were in general much stronger than the corresponding phenotypic correlations (Tables 6 and 7). Specifically, as already established for these data by Cheng et al. [9], average daily feed intake had moderately high genetic correlation estimates with health score in the finisher and with treatment rates but a low estimate with mortality. In contrast, feed intake duration and number of visits had moderately high genetic correlation estimates with mortality but low estimates with health scores and treatment rates. This indicates that genetically, mortality was more associated with feed intake duration and number of visits, while treatment rates and health scores were more related with feed intake. Water dispensed, duration, and number of visits generally had moderately high genetic correlation estimates with treatment rates and mortality. It is noteworthy that water dispensed had stronger genetic correlation estimates with treatment and mortality rates than water disappearance, which means that, genetically, water dispensed was more affected by disease than water disappearance.

Day-to-day variation in feed intake had moderate to high genetic correlation estimates with mortality across the nursery and finisher (Table 6), which was already reported for these data by Putz et al. [8] and Cheng et al. [9]. Day-to-day variation for feed intake duration and number of visits, on the other hand, had moderately high genetic correlation estimates with health scores, which were not reported in these previous studies. These moderate to high genetic correlation estimates were in the expected direction (negative with health score and positive with mortality), which suggests that these traits are potential indicator traits to select for disease resilience. Unfortunately, although day-to-day variation for water intake duration and number of visits had moderately high genetic correlation estimates with treatment rates, the negative sign of these estimates was opposite to expections. One of the reasons may be that healthy pigs are more active, developing thirst, or play with the drinker more than sick pigs. The proportion of off-days for feed intake had moderately high genetic correlation estimates with health score in the finisher and with treatment and mortality rates, and these estimates were in the expected directions. Proportions of off-days for feed intake duration and number of visits, however, only had moderately high genetic correlation estimates with mortality. Interestingly, the proportion of off-days for feed intake rate was estimated to be moderately genetically correlated with health score in the finisher and with treatment rates. The proportion of off-days for some drinking traits was also estimated to be moderately genetically correlated with clinical traits but, again, the signs of these estimates were opposite to expectations. Generally speaking, there were very few feeding or drinking traits that showed consistent moderate or strong genetic correlations with all clinical disease traits. This is somewhat unexpected because strong genetic correlations were reported among these three clinical traits [9].

### Resilience indicator traits based on feeding and drinking traits

Clinical disease traits such as treatment and mortality rates have been shown to have low heritability, both in the data used here [9] and in the literature [31, 32] and are thus, difficult to improve by direct selection. Therefore, an important aim of this study was to explore indicator traits of disease resilience that can be selected on instead. To be a useful indicator trait for indirect selection for disease resilience, it must be moderately to highly heritable and have a high genetic correlation with disease resilience. Based on the same data as used here, Putz et al. [8] and Cheng et al. [9] suggested that day-to-day variation and the proportion of off-days for feed intake and duration had great potential as indicators because of their moderately high heritabilities and strong genetic correlations with treatment and mortality rates. In the present study, additional feeding and drinking traits were investiged and some of these showed great potential as well. The number of feed intake visits was highly heritable (0.51) and moderately genetically correlated with mortality in the finisher and, thus, could be used as an indicator trait to select against mortality under disease. Water intake duration and number of visits also had high estimates of heritability (0.54 and 0.58, respectively) and moderate genetic correlations with treatment and mortality rates, especially for treatment rate across the nursery and finisher (− 0.39 to − 0.45). Hence, these two drinking traits also have great potential as indicator traits to select for disease resilience. The water-to-feed ratio traits, obtained by fitting the corresponding feeding traits as covariates when analyzing drinking traits, are also promising because of their even higher estimates of heritability (0.56 to 0.60) and similar magnitudes of genetic correlations with clinical traits as the corresponding non-ratio drinking traits.

Electronic feeders have been implemented in most nucleus breeding programs and Harlizius et al. [33] has shown that day-to-day variation in individual feed intake collected on high-health nucleus farms can be used to improve finisher survival rate. Electronic feeders could also be used in commercial farms to provide the data needed to implement these indicator traits. With the availability of new technologies such as 3D cameras, thermal imaging, and sensors, recording feeding and drinking behavior traits on commercial farms can be implemented. Incorporation of these indicator traits into breeding programs could result in genetic improvement of resilience to disease. To achieve this goal, a specialized disease challenge facility or one or more commercial farms with severe disease challenges could be used for collection of these resilience indicator traits and used to predict breeding values for selection candidates in the nucleus by genomic prediction.

## Conclusions

Drinking and feeding traits under a severe polymicrobial disease challenge in general had high estimates of heritability, especially for duration and number of visits for both feed and water intake. Phenotypic and genetic correlations among feeding traits were generally low under the disease challenge but drinking traits showed high correlations amongst each other. Corresponding feeding and drinking traits were not strongly correlated with each other under the disease challenge conditions. Drinking traits generally had low genetic correlations with growth rate under challenge but some feeding traits such as daily feed intake and intake rate had moderate to strong genetic correlations with growth rate in the finisher. Day-to-day variation and the proportion of off-days for drinking traits were not highly genetically correlated with treatment and mortality rates, in contrast to the corresponding feeding traits. However, water intake duration and number of visits were highly heritable and had moderately high genetic correlations with treatment and mortality rates and, thus, are potential indicator traits to select for disease resilience. Especially promising is the number of water intake visits, which had a high heritability of 0.58 and moderately high genetic correlations of − 0.45 with treatment rate and − 0.19 with mortality.

## Supplementary Information

**Additional file 1: Fig. S1**. Custom-made individual water intake recording system, consisting of a 3 L bowl, closed on 3 sides to reduce waste, with a nipple that can be activated by the pig, and an in-line water meter, as well as a water level meter for the bowl. The system allows for each visit by an individual pig, identified by radio frequency tags, the recording of time of day, duration, and water disappearance from the nipple and from the bowl. **Fig. S2**. Raw daily feed intake (FI) and water disappearance (WI) for a randomly selected animal (0132). Both WI and FI had large day-to-day variation and had concurrent drops at around 120 and 160 days. **Fig. S3**. Raw daily feed intake duration (FIDUR) and water intake duration (WIDUR) for a randomly selected animal (0132). **Fig. S4**. Raw daily feed intake visits (FInVisits) and water intake visits (WInVisits) for a randomly selected animal (0132). **Fig. S5**. Raw daily feed intake rate (FIRT) and water disappearance rate (WIRT) for a randomly selected animal (0132). **Fig. S6**. Raw water disappearance (WI) and predicted water disappearance (WI_RR) patterns defined using quadratic random regression model for for individual pigs in batch 1A. **Fig. S7**. Histogram for average daily water disappearance (ADWI) and water dispensed (ADWD). **Fig. S8**. Raw daily water disappearance (WI), number of visits (WInVisits), and duration (WIDUR) for a randomly selected animal (0159).

## Data Availability

Data used are on commercially owned animals and are, therefore, not publicly available. Data are, however, available from the authors upon reasonable request.

## Abbreviations

ADFI: Average daily feed intake
ADG: Average daily gain
ADWI: Average daily water disappearance
ADWD: Average daily water dispensed
ADWI|ADFI: Ratio trait by fitting ADFI as covariate for ADWI
ADWD|ADFI: Ratio trait by fitting ADFI as covariate for ADWD
ADFI|ADWI: Ratio trait by fitting ADWI as covariate for ADFI
ADFI|ADWD: Ratio trait by fitting ADWD as covariate for ADFI
CV: Coefficient of variation
FIDUR: Average daily feed intake duration
FInVisits: Average number of daily feed intake visits
FIRT: Average daily feed intake rate
FIDUR|WIDUR: Aatio trait by fitting WIDUR as covariate for FIDUR
FInVisits|WInVisits: Aatio trait by fitting WInVisits as covariate for FInVisits
FinADG: Average daily gain in finisher
FinMOR: Mortality rate for pigs in finisher
FinTRT: Number of health treatments per pig in finisher
GBLUP: Genomic best linear unbiased prediction
IQR: Interquartile range
IQRP: Predicted IQR
OFF_FIDUR_: Proportion of off-feed days for feed intake duration
OFF_FI_: Proportion of off-feed days for feed intake
OFF_FInVisits_: Proportion of off-feed days for number of feed intake visits
OFF_FIRT_: Proportion of off-feed days for feed intake rate
OFF_WI_: Proportion of off-water days for water disappearance
OFF_WD_: Proportion of off-water days for water dispensed
OFF_WIDUR_: Proportion of off-water days for water intake duration
OFF_WInVisits_: Proportion of off-water days for water intake visits
OFF_WIRT_: Proportion of off-water days for water disappearance rate
OFF_WDRT_: Proportion of off-water days for water dispense rate
SNP: Single nucleotide polymorphism
VAR_FIDUR_: Day-to-day variation in feed intake duration
VAR_FI_: Day-to-day variation in feed intake
VAR_FInVisits_: Day-to-day variation in number of feed intake visits
VAR_FIRT_: Day-to-day variation in feed intake rate
VAR_WI_: Day-to-day variation in water disappearance
VAR_WD_: Day-to-day variation in water dispensed
VAR_WIDUR_: Day-to-day variation in water intake duration
VAR_WInVisits_: Day-to-day variation in number of water intake visits
VAR_WIRT_: Day-to-day variation in water disappearance rate
VAR_WDRT_: Day-to-day variation in water dispense rate
WIDUR: Average daily water intake duration
WInVisits: Average number of daily water intake visits
WIRT: Average daily water disappearance rate
WDRT: Average daily water dispensed rate
WIDUR|FIDUR: Ratio trait by fitting FIDUR as covariate for WIDUR
WInVisits|FInVisits: Ratio trait by fitting FInVisits as covariate for WInVisits
