# Supplementary material for: Genetic parameters of drinking and feeding traits of wean-to-finish pigs under a polymicrobial natural disease challenge
Source: J Anim Sci Biotechnol. 2021 Sep 8;12:105. doi: 10.1186/s40104-021-00622-x (PMC8425002; doi:10.1186/s40104-021-00622-x)
Supplement: Supplementary file 1 — Additional file 1: Fig. S1. Custom-made individual water intake recording system, consisting of a 3 L bowl, closed on 3 sides to reduce waste, with a nipple that can be activated by the pig, and an in-line water meter, as well as a water level meter for the bowl. The system allows for each visit by an individual pig, identified by radio frequency tags, the recording of time of day, duration, and water disappearance from the nipple and from the bowl. Fig. S2. Raw daily feed intake (FI) and water disappearance (WI) for a randomly selected animal (0132). Both WI and FI had large day-to-day variation and had concurrent drops at around 120 and 160 days. Fig. S3. Raw daily feed intake duration (FIDUR) and water intake duration (WIDUR) for a randomly selected animal (0132). Fig. S4. Raw daily feed intake visits (FInVisits) and water intake visits (WInVisits) for a randomly selected animal (0132). Fig. S5. Raw daily feed intake rate (FIRT) and water disappearance rate (WIRT) for a randomly selected animal (0132). Fig. S6. Raw water disappearance (WI) and predicted water disappearance (WI_RR) patterns defined using quadratic random regression model for for individual pigs in batch 1A. Fig. S7. Histogram for average daily water disappearance (ADWI) and water dispensed (ADWD). Fig. S8. Raw daily water disappearance (WI), number of visits (WInVisits), and duration (WIDUR) for a randomly selected animal (0159). [file 40104_2021_622_MOESM1_ESM.docx]

**Supplementary figures:**

**
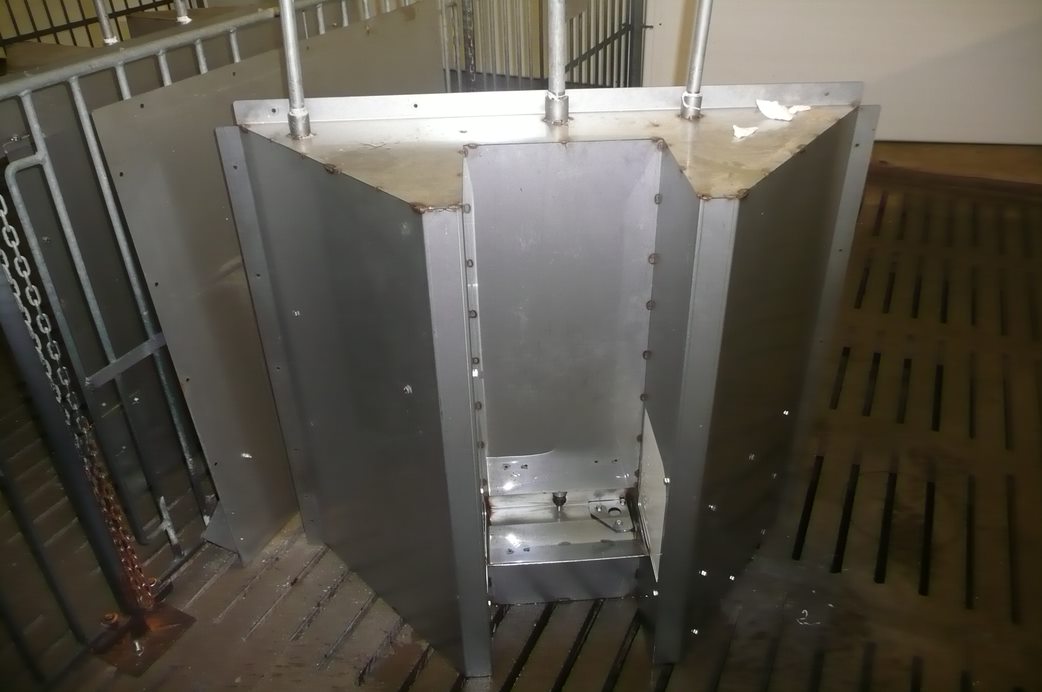
**

Figure S1. Custom-made individual water intake recording system, consisting of a 3 L bowl, closed on 3 sides to reduce waste, with a nipple that can be activated by the pig, and an in-line water meter, as well as a water level meter for the bowl. The system allows for each visit by an individual pig, identified by radio frequency tags, the recording of time of day, duration, and water disappearance from the nipple and from the bowl.

**
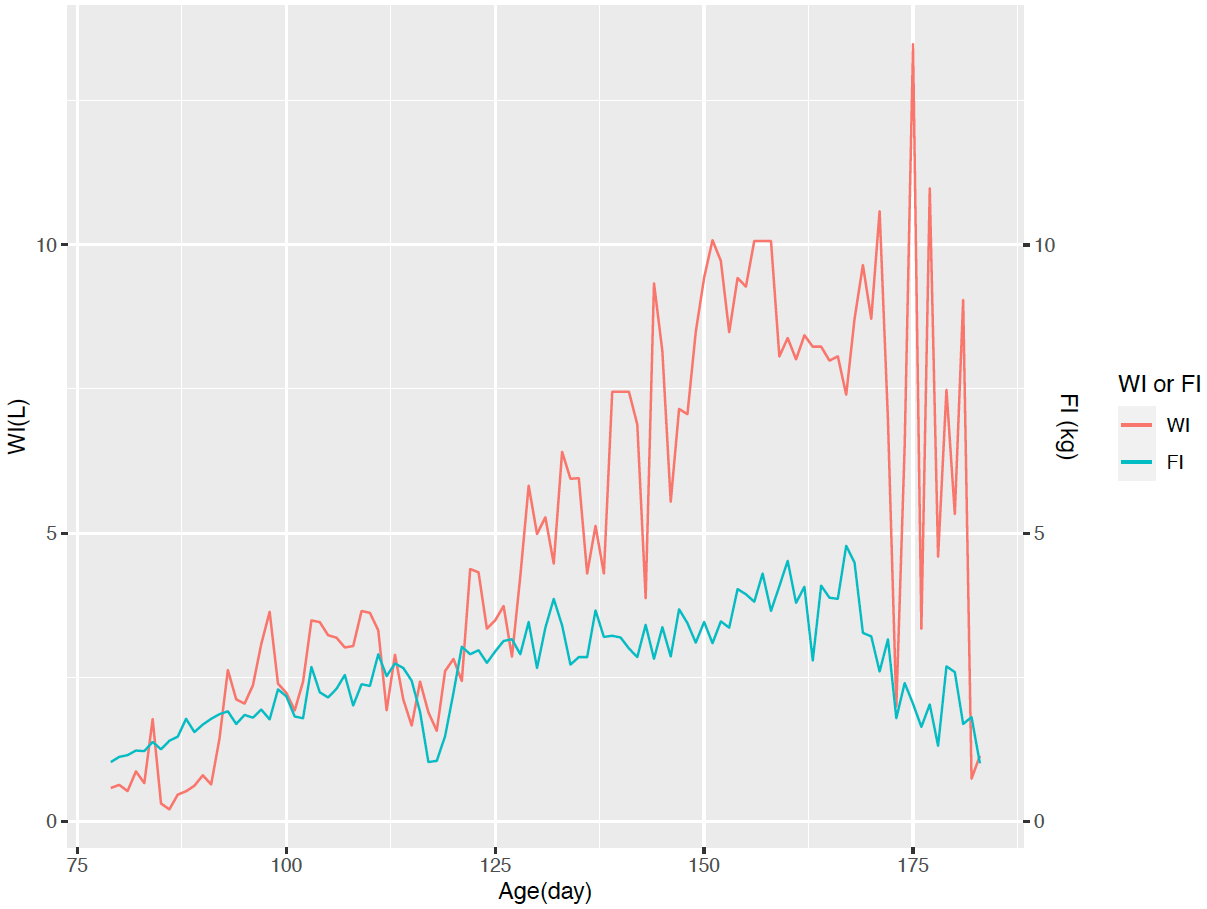
**

Figure S2. Raw daily feed intake (FI) and water disappearance (WI) for a randomly selected animal (0132). Both WI and FI had large day-to-day variation and had concurrent drops at around 120 and 160 days.

**
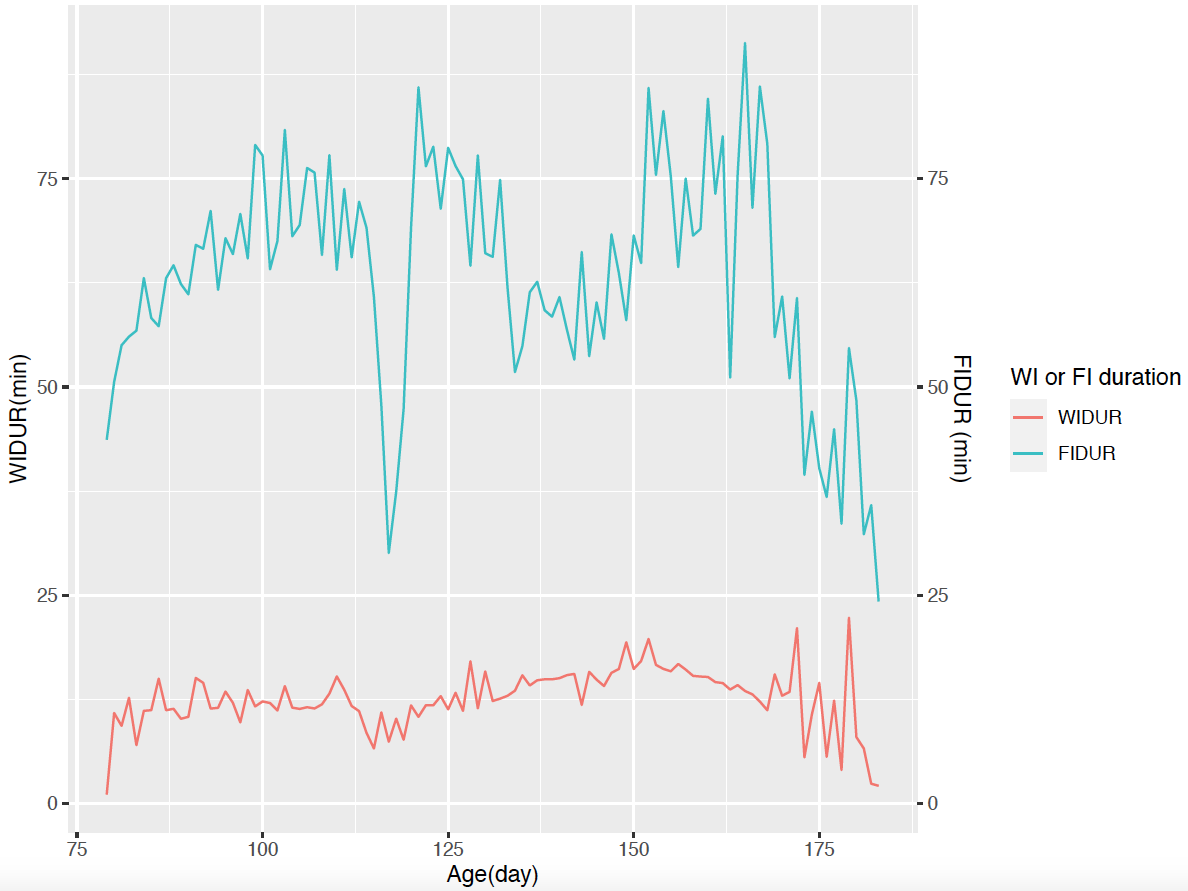
**

Figure S3. Raw daily feed intake duration (FIDUR) and water intake duration (WIDUR) for a randomly selected animal (0132).

**
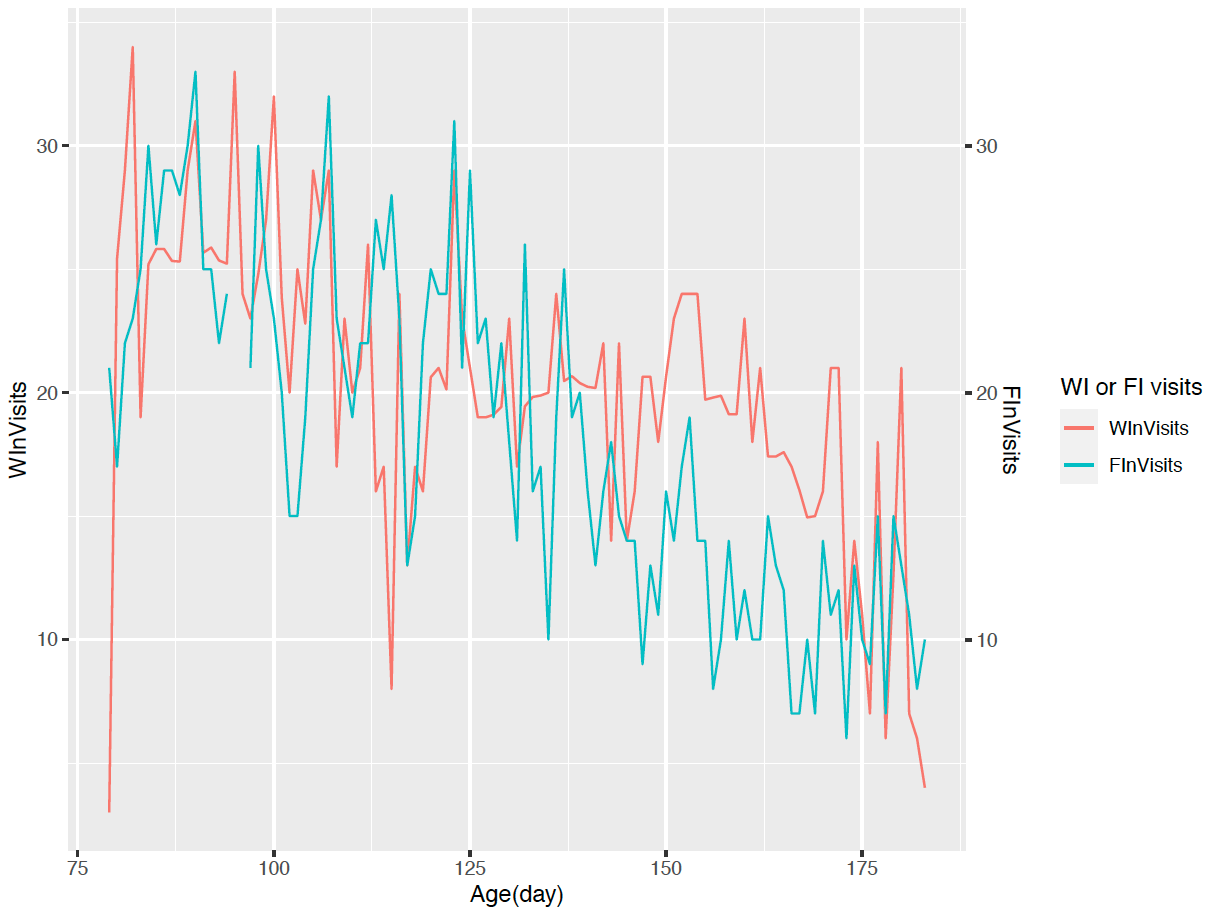
**

Figure S4. Raw daily feed intake visits (FInVisits) and water intake visits (WInVisits) for a randomly selected animal (0132).

**
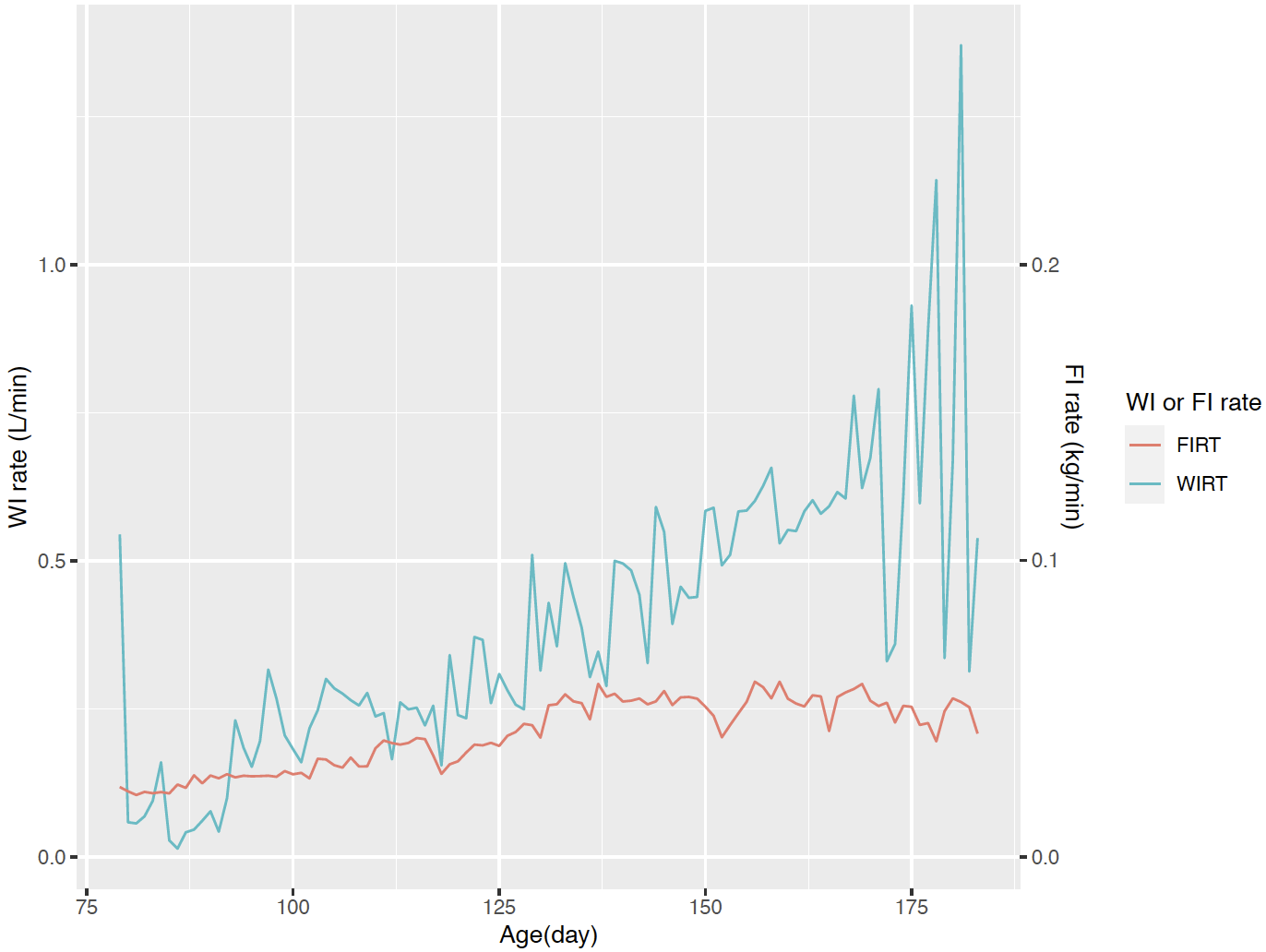
**

Figure S5. Raw daily feed intake rate (FIRT) and water disappearance rate (WIRT) for a randomly selected animal (0132).

**
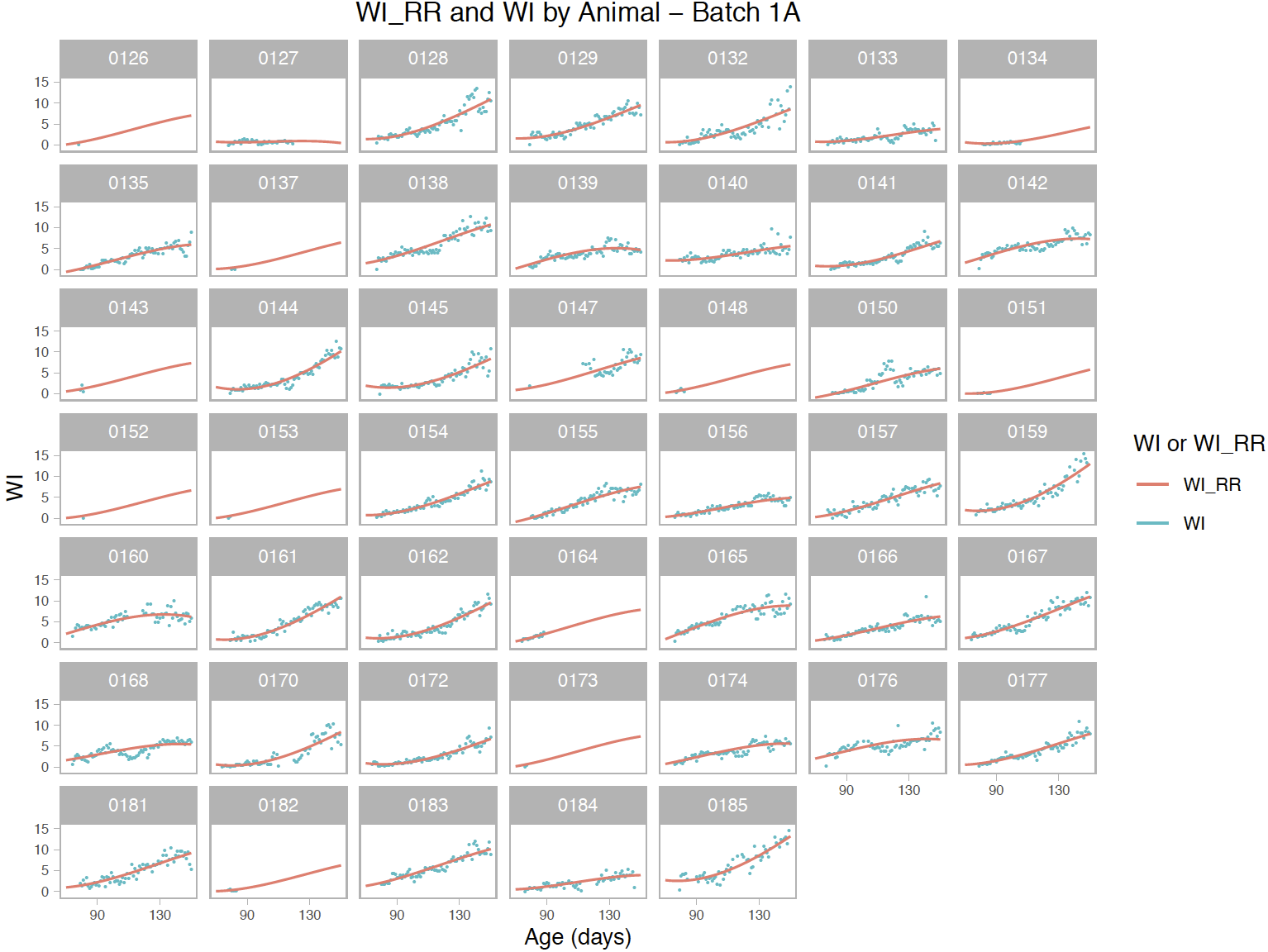
**

Figure S6. Raw water disappearance (WI) and predicted water disappearance (WI_RR) patterns defined using quadratic random regression model for for individual pigs in batch 1A.

**
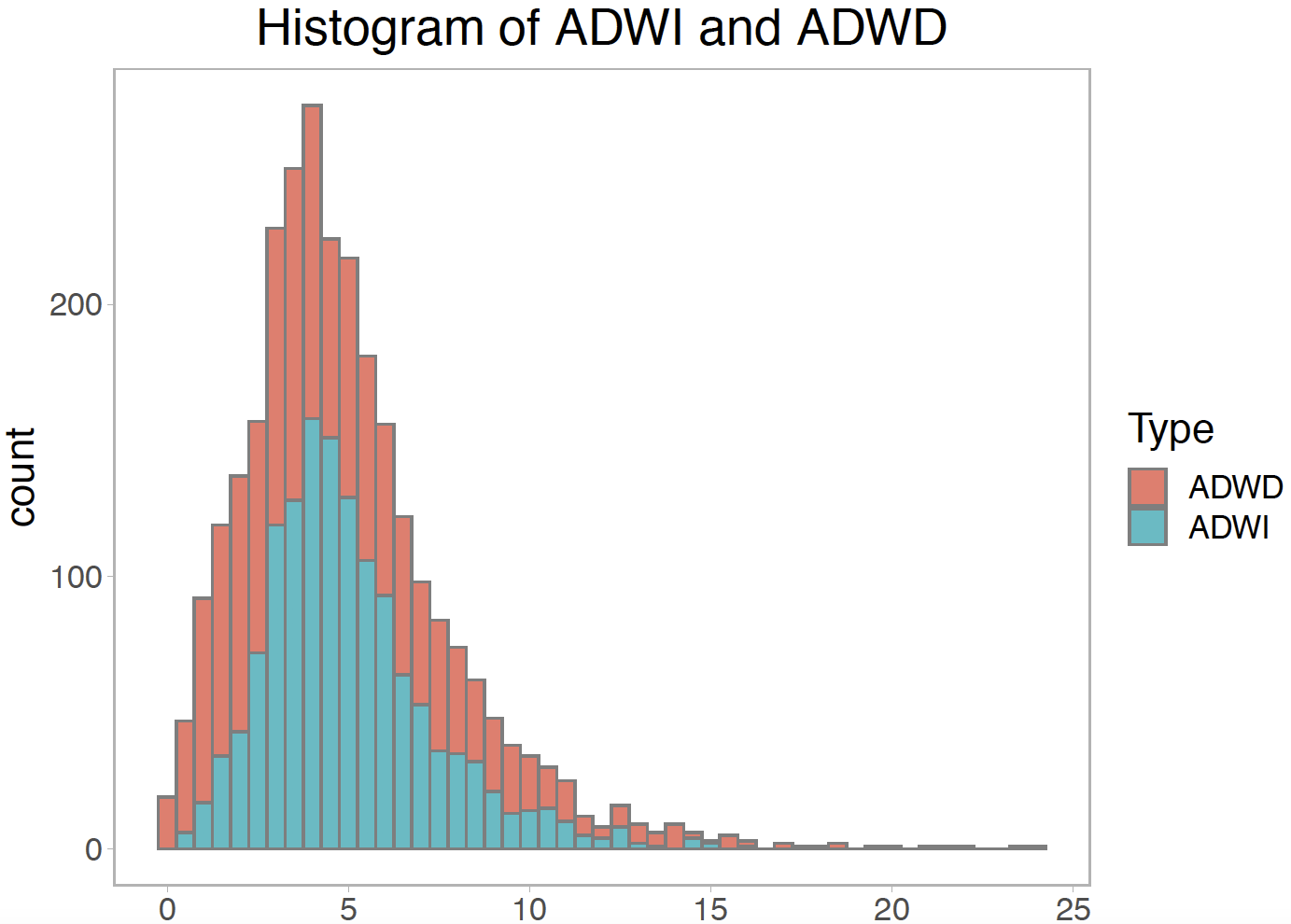
**

Figure S7. Histogram for average daily water disappearance (ADWI) and water dispensed (ADWD).

**
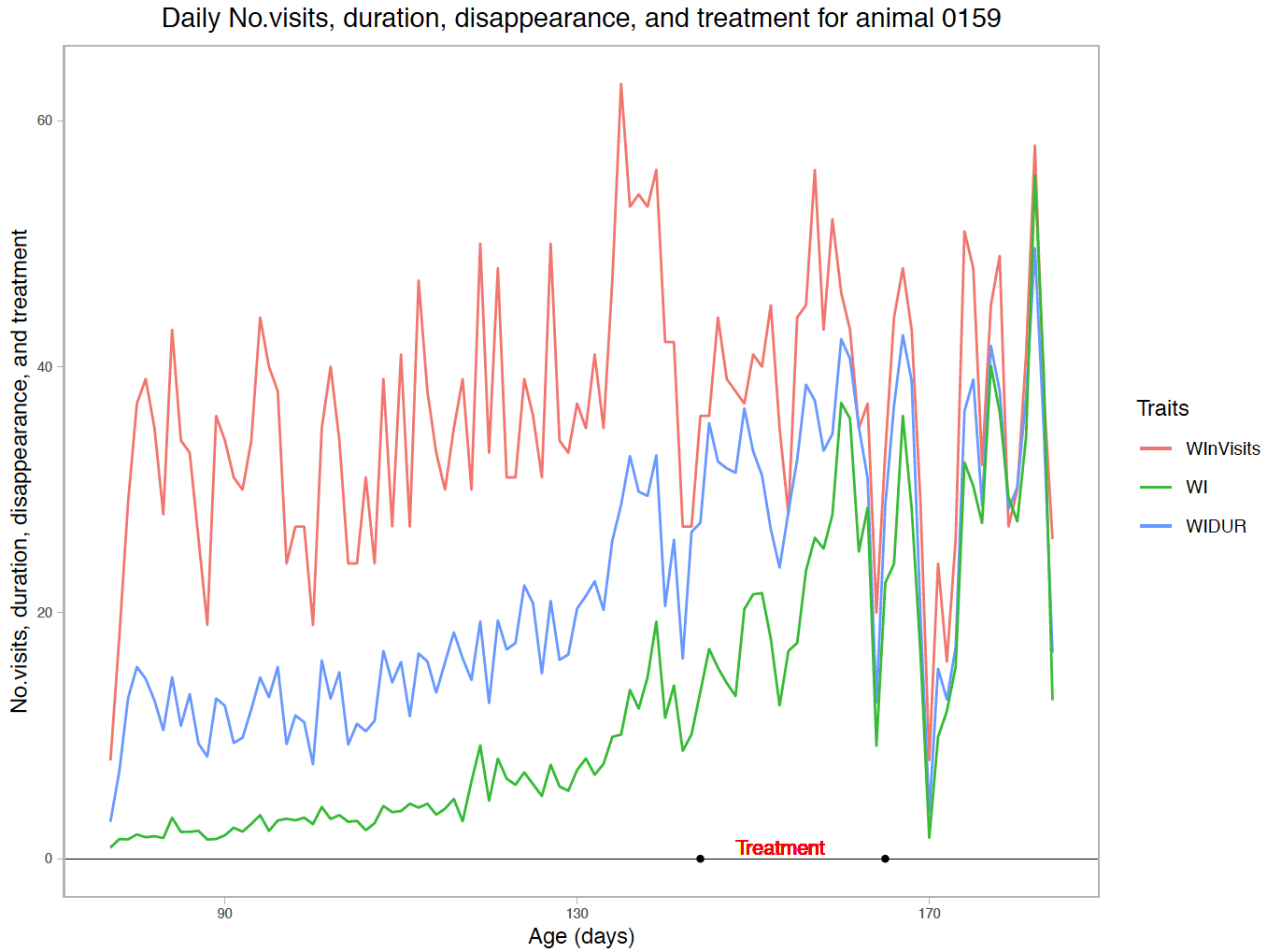
**

Figure S8. Raw daily water disappearance (WI), number of visits (WInVisits), and duration (WIDUR) for a randomly selected animal (0159).
